# Bioinformatic analysis of defective viral genomes in SARS-CoV-2 and its impact on population infection characteristics

**DOI:** 10.3389/fimmu.2024.1341906

**Published:** 2024-01-29

**Authors:** Zhaobin Xu, Qingzhi Peng, Jian Song, Hongmei Zhang, Dongqing Wei, Jacques Demongeot, Qiangcheng Zeng

**Affiliations:** ^1^ Department of Life Science, Dezhou University, Dezhou, China; ^2^ State Key Laboratory of Microbial Metabolism, Shanghai-Islamabad-Belgrade Joint Innovation Center on Antibacterial Resistances, Shanghai Jiao Tong University, Shanghai, China; ^3^ Joint International Research Laboratory of Metabolic & Developmental Sciences and School of Life Sciences and Biotechnology, Shanghai Jiao Tong University, Shanghai, China; ^4^ Zhongjing Research and Industrialization Institute of Chinese Medicine, Zhongguancun Scientific Park, Meixi, Nanyang, Henan, China; ^5^ Peng Cheng National Laboratory, Shenzhen, Guangdong, China; ^6^ Laboratory AGEIS EA 7407, Team Tools for e-Gnosis Medical, Faculty of Medicine, University Grenoble Alpes (UGA), F-38700 La Tronche, France

**Keywords:** SARS-CoV-2, defective viral genome, semi-infectious particle, virus evolution, genome coverage, population infection characteristics, mathematical modeling

## Abstract

DVGs (Defective Viral Genomes) are prevalent in RNA virus infections. In this investigation, we conducted an analysis of high-throughput sequencing data and observed widespread presence of DVGs in SARS-CoV-2. Comparative analysis between SARS-CoV-2 and diverse DNA viruses revealed heightened susceptibility to damage and increased sequencing sample heterogeneity within the SARS-CoV-2 genome. Whole-genome sequencing depth variability analysis exhibited a higher coefficient of variation for SARS-CoV-2, while DVG analysis indicated a significant proportion of recombination sites, signifying notable genome heterogeneity and suggesting that a large proportion of assembled virus particles contain incomplete RNA sequences. Moreover, our investigation explored the sequencing depth and DVG content differences among various strains. Our findings revealed that as the virus evolves, there is a notable increase in the proportion of intact genomes within virus particles, as evidenced by third-generation sequencing data. Specifically, the proportion of intact genome in the Omicron strain surpassed that of the Delta and Alpha strains. This observation effectively elucidates the heightened infectiousness of the Omicron strain compared to the Delta and Alpha strains. We also postulate that this improvement in completeness stems from enhanced virus assembly capacity, as the Omicron strain can promptly facilitate the binding of RNA and capsid protein, thereby reducing the exposure time of vulnerable virus RNA in the host environment and significantly mitigating its degradation. Finally, employing mathematical modeling, we simulated the impact of DVG effects under varying environmental factors on infection characteristics and population evolution. Our findings provide an explanation for the close association between symptom severity and the extent of virus invasion, as well as the substantial disparity in population infection characteristics caused by the same strain under distinct environmental conditions. This study presents a novel approach for future virus research and vaccine development.

## 1 Introduction

The emergence of SARS-CoV-2 has posed a persistent and significant threat to human health (1–3), challenging conventional academic paradigms, including the classical concept of herd immunity (4–6). Despite substantial research efforts, numerous enigmatic aspects of this coronavirus remain unresolved. These include the notable variations in population infection characteristics, which have been linked to climate conditions and control strategies (7). Scientific evidence supports the efficacy of masks as preventive measures, not only in reducing infection rates but also in mitigating the severity of illness following exposure (8, 9). Furthermore, the implementation of stringent control measures has demonstrated a capacity to reduce the incidence of severe cases, as exemplified by the COVID-19 trend in China. For instance, during the spring outbreak in Shanghai in 2022, over 94% of infected individuals presented with mild or asymptomatic symptoms (9), whereas this proportion significantly declined during the nationwide Omicron surge at the end of 2022. The influence of climate on symptom manifestation is another critical consideration. Notably, the extensive Omicron outbreak in China towards the end of 2022 revealed a higher prevalence of severe symptoms among individuals in northern regions, while those infected in southern regions exhibited a markedly elevated proportion of asymptomatic or mild cases in comparison to their northern counterparts. Additionally, lower mortality rates observed in tropical areas such as Singapore, as opposed to Europe and North America (10), despite infections from identical or closely related viral strains, suggest underlying factors beyond medical interventions. This underscores the need for a deeper understanding of the observed variations in population infection characteristics. Through the application of bioinformatics analysis and mathematical simulations, our research uniquely attributes these distinctions to viral heterogeneity, specifically the presence of defective viral genomes (DVGs).

Defective viral genomes (DVGs) are truncated, structurally distinct RNA molecules that emerge during the replication of RNA viruses, commonly observed in viral infections (11–13). The existence of DVGs was initially identified by Preben Von Magnus in the late 1940s as incomplete influenza viruses capable of hindering the replication of wild-type viruses (14). Subsequently, Alice Huang and David Baltimore introduced the term “defective interfering” (DI) particles or DIPs to describe viral particles containing regular structural proteins but only a fraction of the viral genome. They further specified that DIPs can only replicate in the presence of a helper virus and impede the intracellular replication of non-defective homologous viruses (15). Although the exact mechanism underlying DVG generation remains incompletely understood, it is widely accepted that RNA polymerase plays a pivotal role in their synthesis (11, 16, 17). RNA polymerase, characterized by its error-prone nature and lack of proofreading activity, is susceptible to errors during genome replication. These errors can result in the production of truncated or defective viral genomes that serve as templates for the generation of DVGs.

Prior investigations utilizing high-throughput sequencing data have revealed the widespread presence of defective viral genomes (DVGs) or semi-infectious particles (SIPs) in RNA viruses (18–21). The existence of DVGs can exert both favorable and adverse impacts on virus replication and pathogenesis. On one hand, DVGs may impede the replication of full-length viral genomes, resulting in diminished viral titers and attenuated virulence. Conversely, DVGs have the potential to trigger the host immune response and augment the production of antiviral cytokines, culminating in heightened viral clearance and enhanced immune protection (11, 22, 23). Recent investigations have also been focused on elucidating the role of DVGs in COVID-19 infection. One avenue of exploration involves leveraging DVGs as antiviral therapeutics, given findings indicating their substantial capacity to inhibit virus replication (24, 25). Sun Yan et al. observed notable variations in the abundance of DVGs across distinct cell types, with the proportion of DVGs exhibiting significant fluctuations over the course of infection (26). Furthermore, they noted a markedly lower abundance of DVGs in asymptomatic individuals compared to symptomatic individuals.

Despite significant progress in recent years regarding the study of viral heterogeneity, several critical questions remain to be addressed. These include: (I) What are the mechanisms underlying the formation of defective viruses? (II) How does viral heterogeneity impact individual infection characteristics? (III) How does viral heterogeneity influence population infection characteristics and viral evolution? To further elucidate these issues, we have employed a comprehensive approach that combines bioinformatics and mathematical modeling. Initially, we verified through viral second- and third-generation sequencing data that errors in RNA replication are the primary mechanism underlying the generation of defective viruses in SARS-CoV-2. Subsequently, utilizing mathematical models, we systematically investigated the impact of viral heterogeneity on individual and population infections. Our models provide an explanation for the enhanced transmissibility of late-stage SARS-CoV-2 variants and the effects of control measures and climate conditions on SARS-CoV-2 infection.

## 2 Materials and methods

### 2.1 Database

In this study, we employed the NCBI virus database (27) as a resource to investigate the genomic information and sequence gaps of various strains. The NCBI Virus database offers a comprehensive collection of viral sequences and associated characteristics, encompassing genome sequences, SRA data, and detailed annotations. Thus, we leveraged this database to extract diverse sequencing results that encompassed genome and SRA information for each strain.

To access the SRA data and reference genome sequences associated with these strains, we utilized the SRA toolbox, a command-line tool developed by the NCBI. This tool facilitated the retrieval of raw sequencing data in the form of SRA files, which were subsequently converted into fastq files for subsequent analysis. These data were then integrated with the corresponding reference genome sequences to obtain a more comprehensive comprehension of the genetic composition of each strain.

### 2.2 Generation of genome depth spectrum and mining of DVGs

In the field of bioinformatics analysis, we have developed a novel algorithm that enables the rapid calculation of sequencing depth. This algorithm requires both the sequencing data and template gene sequences. With this approach, we can quickly calculate the short sequences in the sequencing data that match the template chain and identify breakpoints that occur during the sequencing process.

In the domain of bioinformatics analysis, we have devised a novel algorithm that facilitates swift computation of sequencing depth. This algorithm necessitates the availability of both the sequencing data and template gene sequences. Through this methodology, we expediently determine the concordance between short sequences in the sequencing data and the template chain, thereby identifying breakpoints that arise during the sequencing process.

In order to enhance program efficiency, we utilized a string-matching method instead of traditional sequence alignment algorithms to compute sequence coverage. If a sequencing fragment completely matches a particular section of the reference genome, every position on this fragment will be counted once. If there are no mutations, deletions, or sequence recombination events present in the sequencing fragment, it will exhibit a uniform sequencing depth. However, when significant non-uniformity of the sequence is present, the sequencing depth will show significant fluctuations. Through this approach, we can identify regions with higher or lower coverage than expected.

To further investigate splicing sites or copy-back recombination sites generated by DVGs, we further employed string matching to identify these breakpoints. Sequencing fragments that completely match the reference genome were excluded. If the 5’ end of a sequencing fragment matches until a particular position, referred to as position N in the reference genome, and if the subsequent sequence (*N+n*) can match again in the original genome, then we consider this position as a breakpoint caused by deletion. Similarly, if the 5’ end of a sequencing fragment matches until a particular position, and if the preceding sequence (*N-n*) can match again in the original genome, then we consider this position as a breakpoint generated by copy-back. To exclude point mutations or deletions that may cause breakpoints, we set the value of n to 5, so only gaps or insertions exceeding five bases are defined as breakpoints caused by defective viruses.

Errors in sequencing are indeed common, especially at individual nucleotide positions. To minimize these errors, we employed several strategies. Firstly, we utilized sequencing data with high genome coverage, ensuring that sequences with weak signals were filtered out. Additionally, to ensure the presence of defective viral genomes (DVGs), we imposed a stricter constraint by considering sequences with an intermediate gap of at least five nucleotides as valid. This criterion helps distinguish between single-nucleotide mutations or sequencing errors, where breakpoints in sequence alignment differ by only one or a few nucleotides, and large-scale nucleotide deletions or copybacks, which are indicative of defective viral genomes.

### 2.3 Mathematical modeling of incomplete viral genome in individual infection

**Figure 1:**
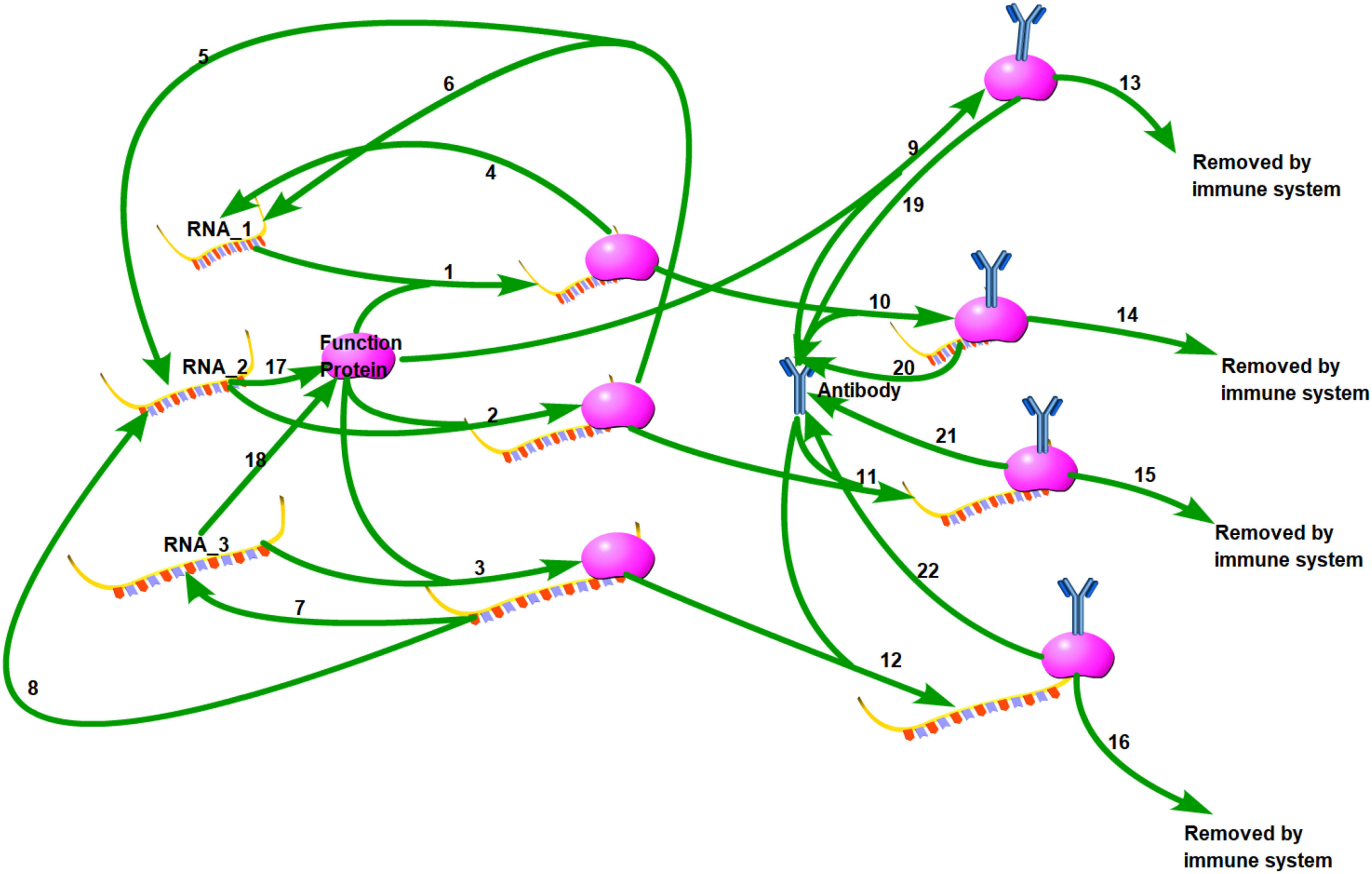
Biochemical scheme of SARS-CoV-2 life cycle. 22 reactions are illustrated in this diagram.

A brief biochemical scheme of the SARS-CoV-2 life cycle is represented in **Figure 1**, with 22 reactions illustrated in **Table 1**. Unlike traditional models, we propose that viruses exhibit polymorphism, meaning that during the replication process, the complete RNA has a small probability of becoming partially deleted due to replication errors made by the polymerase. Our model is based on our previous antibody kinetics model, and therefore, for parameters related to antibody properties, we utilized data from reference (28). The values of k_7_, k_9_, k_11_, and k_13_ were validated using population-level antibody data and vaccine efficacy data over time. Additionally, for parameters k_1_ and k_12_, which are involved in RNA-protein binding and RNA degradation processes, respectively, we referred to data from reference (29). The initial values for these parameters were derived from experimental data. A novel aspect of our model is the consideration of differences in replication and translation of RNAs of different lengths. In practice, these deletions are continuous, but for simplicity in our study, we categorize them into three levels: intact RNA with no deletions (RNA_3), partially deleted RNA (RNA_2), and severely defective RNA (RNA_1). RNA_3 has the longest RNA chain and therefore has the longest replication cycle and the slowest replication rate, which we assume to be 1. However, there is a small probability (assumed to be 10%) that it may transform into RNA_2 during replication (k_5_ = 0.1, k_6_ = 0.9). RNA_2 has a shorter base length and a faster replication rate of 1.2. Similarly, there is a 1% probability of RNA_2 transforming into RNA_1 during replication (k_3_ = 0.12, k_4 _= 1.08). Additionally, we note that RNA_1 has lost the ability to generate functional viral proteins due to severe internal base deletions, while the translated protein activity of RNA_2 is partially impaired. Hence, we represent this weaker translation efficiency with k_9_ = 0.5. The translation efficiency of intact RNA_3 is 1 (k_10 _= 1). Although the likelihood of further deletions during virus replication is low, the shorter defective viral genomes have a faster replication rate and their proportion may significantly increase with each replication cycle.

**Table 1:** Reaction index and the name of each reaction in our mathematical model.

Reaction index	Reaction
1	RNA_1 + F k_1_ 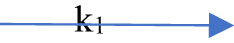 RNA_1-replication complex
2	RNA_2 + F k_1_ 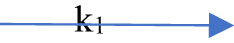 RNA_2-replication complex
3	RNA_3 + F k_1_ 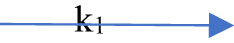 RNA_3-replication complex
4	RNA_1-replication complex k_2_ 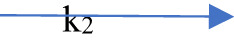 RNA_1
5	RNA_2-replication complex k_3_ 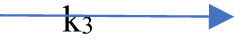 RNA_2
6	RNA_2-replication complex k_4_ 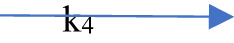 RNA_1
7	RNA_3-replication complex k_5_ 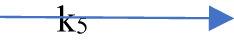 RNA_3
8	RNA_3-replication complex k_6_ 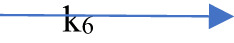 RNA_2
9	F + Antibody k_7_ 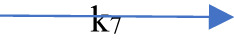 F-antibody complex
10	RNA_1-replication complex + Antibody k_7_ 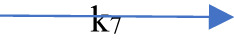 RNA_1-replication -Antibody complex
11	RNA_2-replication complex + Antibody k_7_ 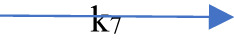 RNA_2-replication -Antibody complex
12	RNA_3-replication complex + Antibody k_7_ 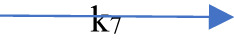 RNA_3-replication -Antibody complex
13	F-antibody complex k_8_ 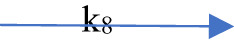 removed by immune system
14	RNA_1-replication -Antibody complex k_8_ 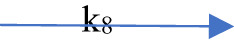 removed by immune system
15	RNA_2-replication -Antibody complex k_8_ 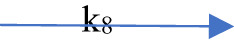 removed by immune system
16	RNA_3-replication -Antibody complex k_8_ 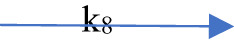 removed by immune system
17	RNA_2 k_9_ 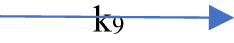 F
18	RNA_3 k_10_ 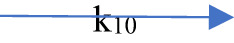 F
19	F-antibody complex k_11_ 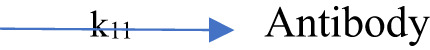 Antibody
20	RNA_1-replication -Antibody complex k_11_ 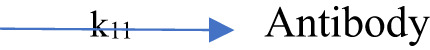 Antibody
21	RNA_2-replication -Antibody complex k_11_ 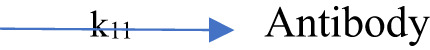 Antibody
22	RNA_3-replication -Antibody complex k_11_ 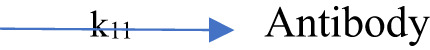 Antibody
23	RNA_1 k_12_ 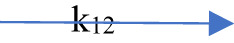 Degradation
24	RNA_2 k_12_ 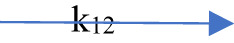 Degradation
25	RNA_3 k_12_ 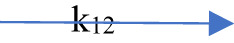 Degradation
26	Antibody k_13_ 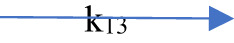 Degradation

The selection of these numerical values did not undergo the traditional process of data fitting, as there was a lack of quantifiable data available for fitting. This may somewhat reduce the reliability of the model. However, our parameter selection follows a consistent pattern. In a homogeneous mixture, the replication rate based on the severely defective RNA (RNA_1) template (1.5) is faster than the replication rate based on the partially deleted RNA (RNA_2) template (1.2). Furthermore, the replication rate based on the RNA_2 template (1.2) is faster than the replication rate based on the intact RNA with no deletions (RNA_3) template (1.0). This is because longer sequences require more time for replication. Additionally, we believe that as the template chain is truncated, the ability to encode functional proteins is affected, or rather, the activity of the encoded functional proteins significantly decreases. For severely defective RNA, functional proteins cannot be translated at all, while for partially deleted RNA (RNA_2), the translation efficiency of functional proteins is impaired (k_9_ = 0.5). As for intact RNA_3, the translation efficiency is highest (k_10_ = 1). Changing these values would affect the specific concentration changes of each component. However, as long as this pattern is maintained, the overall trend and conclusions will remain unchanged.

The ordinary differential equations of the single-agent model are listed in Equations (1–12). The definitions of variables are listed in **Table 2**. The parameters are defined in **Table 3**.

**Table 2:** Time-dependent variables of the mathematical model characterizing the SARS-CoV-2 life cycle.

Variables	Meaning	Initial value
x1	Defective RNA	0
x2	Semi-infectious RNA	0
x3	Full length RNA	1
x4	Functional Protein	0
x5	RNA_1-replication complex	0
x6	RNA_2-replication complex	0
x7	RNA_3-replication complex	0
x8	Antibody	1
x9	F-protein-antibody complex	0
x10	RNA_1-replication-Antibody complex	0
x11	RNA_2-replication-Antibody complex	0
x12	RNA_3-replication-Antibody complex	0

**Table 3:** Estimates of the calibrated model parameters.

Parameter Name	Description	Value	Range,refs
k_1_	Rate of Assembly constant between RNA and viral proteins	5.0e-4	(28, 29)
k_2_	Replication rate of RNA_1(defective viral genome) based on RNA_1-replication complex	1.5	Assumed
k_3_	Replication rate of RNA_1(defective viral genome) based on RNA_2-replication complex	0.12	Assumed
k_4_	Replication rate of RNA_2(semi-infectious viral genome) based on RNA_2-replication complex	1.08	Assumed
k_5_	Replication rate of RNA_2(semi-infectious viral genome) based on RNA_3-replication complex	0.1	Assumed
k_6_	Replication rate of RNA_3(full viral genome) based on RNA_3-replication complex	0.9	Assumed
k_7_	Antibody binding constant	5.0e-4	(28)
k_8_	Clearing rate of antibody-antigen complex	0.5	(28)
k_9_	Translation rate of viral functional proteins based on semi-infectious RNA template	0.5	Assumed
k_10_	Translation rate of viral functional proteins based on full-length RNA template	1	Assumed
k_11_	Activation constant of antigen-antibody complex on antibody regeneration	0.9	(28)
k_12_	RNA degradation rate	0.01	(29)
k_13_	Antibody degradation rate	0.01	(28)


(1) $$\frac{\text{d}\left(x1\right)}{\text{d}t}=-k1\;*\;x1\;*\;x4+k2\;*\;x5+k4\;*\;x6-k12\;*\;x1;$$

(2) $$\frac{d\left(x2\right)}{dt}=-k1\;*\;x2\;*\;x4+k3\;*\;x6+k6\;*\;x7-k12\;*\;x2;$$

(3) $$\frac{d\left(x3\right)}{dt}=-k1\;*\;x3\;*\;x4+k5\;*\;x7-k12\;*\;x3;$$

(4) $$\frac{d\left(x4\right)}{dt}=-k1\;*\;\left(x1+x2+x3\right)\;*\;x4+k9\;*\;x2+k10\;*\;x3-k7\;*\;x4\;*\;x8;$$

(5) $$\frac{d\left(x5\right)}{dt}=k1\;*\;x1\;*\;x4-k7\;*\;x5\;*\;x8;\;$$

(6) $$\frac{d\left(x6\right)}{dt}=k1\;*\;x2\;*\;x4-k7\;*\;x6\;*\;x8;\;$$

(7) $$\frac{d\left(x7\right)}{dt}=k1\;*\;x3\;*\;x4-k7\;*\;x7\;*\;x8;$$

(8) $$\frac{d\left(x8\right)}{dt}=-k7\;*\;\left(x4+x5+x6+x7\right)\;*\;x8+k11\;*\;\left(x9+x10+x11+x12\right)-k13\;*\;x8;$$

(9) $$\frac{d\left(x9\right)}{dt}=k7\;*\;x4\;*\;x8-k8\;*\;x9;$$

(10) $$\frac{d\left(x10\right)}{dt}=k7\;*\;x5\;*\;x8-k8\;*\;x10;$$

(11) $$\frac{d\left(x11\right)}{dt}=k7\;*\;x6\;*\;x8-k8\;*\;x11;$$

(12) $$\frac{d\left(x12\right)}{dt}=k7\;*\;x7\;*\;x8-k8\;*\;x12;$$

### 2.4 Mathematical modeling of incomplete viral genome in population infection

We proceed to incorporate the virus-antibody interaction within our agent-based model. In our previous study (30), we introduced a continuous Markov-chain model for simulating epidemics. This model considers a population consisting of N individuals, each exhibiting varying contact probabilities with others. The probability of infection is directly proportional to the contact probability, with the infection probability being equal to the contact probability itself. Notably, the contact probability between an individual and themselves is assigned a value of zero. Consequently, a matrix of size N × N is constructed, exhibiting the following properties:


(13) $$\;M_{interaction}\left(i,i\right)=\;0\;$$

(14) $$\;M_{interaction}\left(i,j\right)=\;M_{interaction}\left(j,i\right)$$

where $M_{interaction}\left(i,j\right)$ stands for the interaction possibility between individual *i* and individual *j*. An accurate contact matrix can be obtained by tracking the individual contact probability in a natural population group. For example, each person’s mobile phone can be recorded to obtain the population contact matrix within a particular time phase. The contact matrix is temporal and dynamic, which means it changes over time. However, it is difficult to obtain such accurate data at present. Therefore, the contact frequency is determined according to the relative distance between individuals, as shown in Equation (15).


(15) $$M_{interaction}\left(i,j\right)\;=\;min\left(c1,\frac{c2}{distance{\left(i,j\right)}^{n}}\right)$$

where c1 is the maximal contact possibility between agent *i* and agent *j*. In particular, the values of c1 , c2 , and *n* are preliminarily determined according to the initial reproduction constant *R_0_* of the virus. According to the contact matrix, we can further determine the number of environmental invasive viruses received by a specific individual in a specific period of time.


$$\;\varphi \left(x_{j}\right)=f\left(x_{j}\right)\;*\;M_{interaction}\left(i,j\right)\;;$$

(16) $$f\left(x_{j}\right)=\;\mu \;x_{j}$$

$\varphi \left(x_{j}\right)$ represents the inhaled virus of individual *i* from infected people *j*. $f\left(x_{j}\right)$ represents the overall released virus from infected person *j.* $f\left(x_{j}\right)$ is positive related to the virus loading $x_{j}$ in corresponding infected agent with a correlation factor μ .

An extensive set of ordinary differential equations is further constructed. Assuming that the number of individuals in the population is *N*, each component is same to the single-agent model.A large set of equations are set in Equations (17-31):


(17) $$\frac{\text{d}\left(x1_{i}\right)}{\text{d}t}=-k1\;*\;x1_{i}\;*\;x4_{i}+k2\;*\;x5_{i}+k4\;*\;x6_{i}-k12\;*\;x1_{i}+\sum_{j=1}^{n}\varphi \left(x1_{j}\right);$$

(18) $$\frac{d\left(x2_{i}\right)}{dt}=-k1\;*\;x2_{i}\;*\;x4_{i}+k3\;*\;x6_{i}+k6\;*\;x7_{i}-k12\;*\;x2_{i}+\sum_{j=1}^{n}\varphi \left(x2_{j}\right);$$

(19) $$\frac{d\left(x3_{i}\right)}{dt}=-k1\;*\;x3_{i}\;*\;x4_{i}+k5\;*\;x7_{i}-k12\;*\;x3_{i}+\sum_{j=1}^{n}\varphi \left(x3_{j}\right);$$

(20) $$\frac{d\left(x4_{i}\right)}{dt}=-k1\;*\;\left(x1_{i}+x2_{i}+x3_{i}\right)\;*\;x4_{i}+k9\;*\;x2_{i}+k10\;*\;x3_{i}-k7\;*\;x4_{i}\;*\;x8_{i};$$

(21) $$\frac{d\left(x5_{i}\right)}{dt}=k1\;*\;x1_{i}\;*\;x4_{i}-k7\;*\;x5_{i}\;*\;x8_{i};$$

(22) $$\frac{d\left(x6_{i}\right)}{dt}=k1\;*\;x2_{i}\;*\;x4_{i}-k7\;*\;x6_{i}\;*\;x8_{i};$$

(23) $$\frac{d\left(x7_{i}\right)}{dt}=k1\;*\;x3_{i}\;*\;x4_{i}-k7\;*\;x7_{i}\;*\;x8_{i};$$

(24) $$\begin{array}{l} \frac{d\left(x8_{i}\right)}{dt}=-k7\;*\;\left(x4_{i}+x5_{i}+x6_{i}+x7_{i}\right)\;*\;x8_{i}+k11\;*\;\left(x9_{i}+x10_{i}+x11_{i}+x12_{i}\right)\\ -k13\;*\;x8_{i}; \end{array}$$

(25) $$\frac{d\left(x9_{i}\right)}{dt}=k7\;*\;x4_{i}\;*\;x8_{i}-k8\;*\;x9_{i};$$

(26) $$\frac{d\left(x10_{i}\right)}{dt}=k7\;*\;x5_{i}\;*\;x8_{i}-k8\;*\;x10_{i};$$

(27) $$\frac{d\left(x11_{i}\right)}{dt}=k7\;*\;x6_{i}\;*\;x8_{i}-k8\;*\;x11_{i};$$

(28) $$\frac{d\left(x12_{i}\right)}{dt}=k7\;*\;x7_{i}\;*\;x8_{i}-k8\;*\;x12_{i};$$

(29) $$\varphi \left(x1_{j}\right)=\;\mu \;*\;x1_{j}\;*\;M_{interaction}\left(i,j\right);$$

(30) $$\varphi \left(x2_{j}\right)=\;\mu \;*\;x2_{j}\;*\;M_{interaction}\left(i,j\right);$$

(31) $$\varphi \left(x3_{j}\right)=\;\mu \;*\;x3_{j}\;*\;M_{interaction}\left(i,j\right);$$

This system of equations contains 12*N variables, where the last term in (Equations 17-19) $\;\sum_{j=1}^{n}\varphi \left(x_{j}\right)$ represents the overall number of viruses transmitted to this individual by other individuals in the whole population. We can generate random kinetic parameters that conform to the real population distribution through the first step of simulation. For such a large system of ordinary differential equations, we can solve it quickly by increasing the step size.

## 3 Results

### 3.1 Gene sequencing depth profile reveals severe heterogeneity in the SARS-CoV-2 genome

The presence of genetic polymorphisms in viral genomes, including defective viral genomes, has long been acknowledged. Nevertheless, there is currently a dearth of systematic research into the impact of defective viruses on viral transmission and population infection dynamics. Various potential causes for genomic defects in the SARS-CoV-2 virus have been identified, with three compelling reasons for their emergence outlined herein. The first pertains to deletions in the UTR regions, a topic previously explored in our published work (31). The second involves genomic fragmentation, likely attributable to the activity of host cell RNAase enzymes. The third reason concerns the generation of defective viruses, which will be redefined in section 3.2, surpassing the understanding established by previous studies. Irrespective of the origins of genomic heterogeneity in the virus, such variations are manifested in sequencing depth profiles. While fluctuations in sequencing depth at distinct loci may be influenced by diverse factors, a distinct contrast between the genome depth profiles of DNA bacteriophages and SARS-CoV-2 underscores a greater degree of genomic heterogeneity in the novel coronavirus. This genomic heterogeneity constitutes a pivotal factor influencing viral population infection characteristics and evolutionary dissemination.

**Figure 2:**
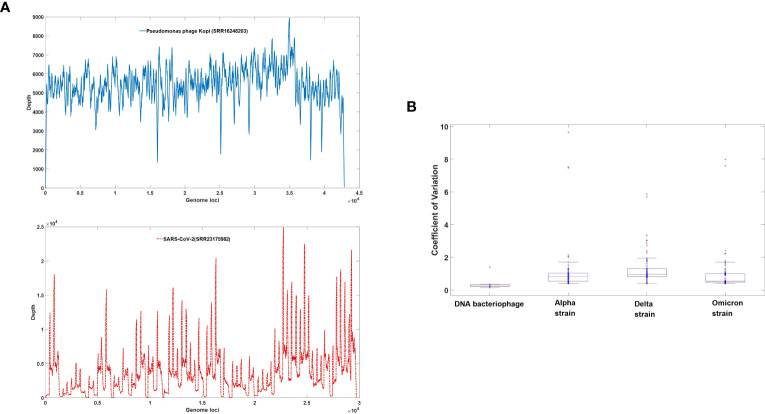
**(A)** Depth coverage of DNA bacteriophage (SRR16248203) and SARS-CoV-2 (SRR23175982). The X-axis represents nucleotide positions, while the Y-axis represents the sequencing depth at each corresponding position. The sequencing depth profiles for the DNA bacteriophage (SRR16248203) sample are plotted as solid blue lines, while the sequencing depth profiles for the SARS-CoV-2 (SRR23175982) sample are represented by dashed red lines. **(B)** Uniformity of NGS data among DNA bacteriophage (**Supplementary Table S1**) and three major strains of SARS-CoV-2. The distribution of the Coefficient of Variation is represented using box plots, with the mean value for each distribution indicated by a red horizontal line.

In this study, we utilized sequencing data from ten types of DNA bacteriophages and 297 second-generation sequencing datasets comprising 99 Alpha strains (**Supplementary Table S2**), 93 Delta strains (**Supplementary Table S3**), and 105 Omicron strains (**Supplementary Table S4**) of SARS-CoV-2. The results are illustrated in **Figure 2**. Specifically, **Figure 2A** provides a comparison of the sequencing depth between a bacteriophage and SARS-CoV-2. The coefficient of variation in gene sequencing depth serves as an effective measure of genome uniformity. As depicted in **Figure 2B**, the coefficient of variation in gene sequencing depth for SARS-CoV-2 is significantly higher than that of DNA bacteriophages, indicating a marked genomic heterogeneity in the novel coronavirus. Noteworthy is the fact that the completeness and uniformity of the genome in omicron virus sequencing surpass those of delta and alpha strains.

### 3.2 Comparison of defective viruses in different SARS-CoV-2 strains

**Figure 3:**
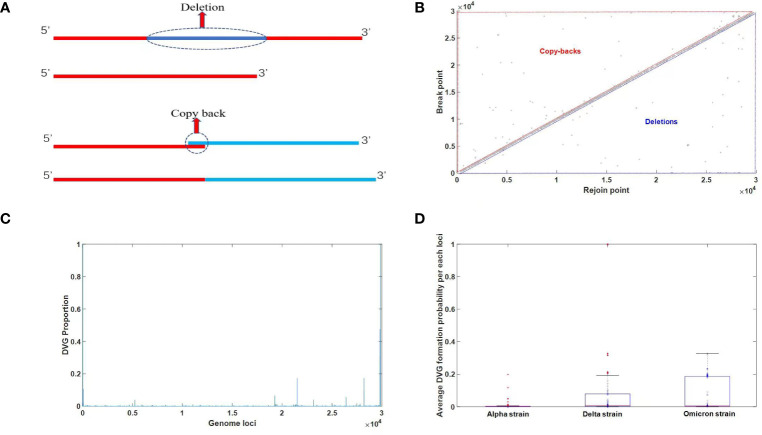
**(A)** A diagram of two types of defective viruses. The term “deletion” refers to the absence of an intermediate segment, while “copy-back” denotes the incorrect recombination resulting from the replication of an intermediate segment, leading to an overall sequence expansion. **(B)** Analysis for junction distribution of DVGs identified in NSG data. The X-axis represents the Rejoin point, while the Y-axis represents the break point. When the sequence number of the Rejoin point is greater than the break point, it indicates a deletion in defective viral genomes (DVGs). Conversely, when the sequence number of the Rejoin point is smaller than the break point, it denotes copy-backs in DVGs. **(C)** Analysis of DVGs transformation probability in each genome loci. The X-axis represents the nucleotide position, while the Y-axis represents the probability of each position becoming a break point or rejoin point, i.e., the probability of forming defective viral genomes (DVGs). **(D)** Comparison of DVGs overall transformation probability among three major strains of SARS-CoV-2. The distribution of the average probability of DVG formation at each nucleotide position for different viral strains is depicted using a box plot, with the mean values indicated by red horizontal lines. The plot reveals consistently low probabilities of DVG formation at individual nucleotide positions across different viral strains, with no significant variation observed.

Conventionally, defective viruses have been defined as viral particles exhibiting partial genomic deletions that significantly impair the function of encoded proteins, leading to an inability to complete replication. These entities are commonly referred to as defective viruses. Notably, no fundamental distinction exists between defective viruses and semi-infectious particles (SIP). When the deleted fragment is relatively short, the virus’s replication function is compromised but not completely abolished, thereby denoted as SIP. In this work, we propose an extended definition for defective viruses, encompassing any modification to the original viral genome, excluding point mutations, that disrupts its normal biological function. This expanded definition considerably broadens the scope of defective viruses. With this foundation, defective viruses can be classified into two categories. The first category pertains to traditionally studied defective viruses, characterized by significant internal genomic deletions. The second category encompasses defective viruses harboring internal duplicated segments. These two scenarios can be visually represented by the pattern diagram depicted in **Figure 3A**.

**Figure 4:**
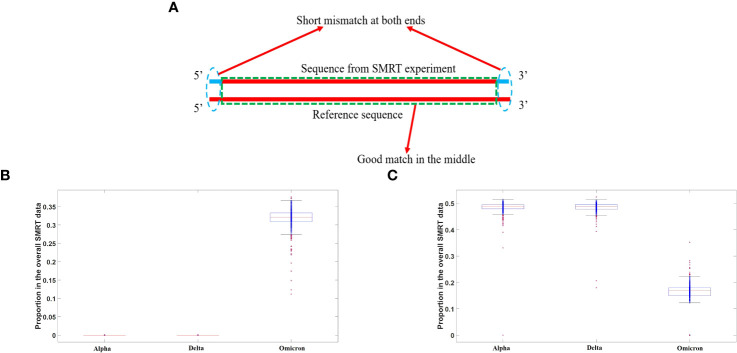
**(A)** A diagram of replication error caused by SARS-CoV-2 RNA polymerase. The majority of mismatches were observed to occur at the two ends of long sequence fragments, as revealed by long-read sequencing using third-generation SMRT technology, as indicated by the dashed blue cycles. **(B)** Proportion of end mutations in the overall SMRT data in three major SARS-CoV-2 strains. The distribution of the proportional probability of mutations at both ends of sequences for different viral strains is depicted using a box plot, with red horizontal lines indicating the average value for each distribution. It can be observed that the proportion for the Omicron strain is significantly lower than that for the Alpha and Delta strains. **(C)** Proportion of full-matched segments in the overall SMRT data in three major SARS-CoV-2 strains. The distribution of the proportion of sequences perfectly matching the reference genome, as determined by third-generation SMRT data, is depicted using a box plot, with red horizontal lines representing the mean value. From the plot, it can be observed that the Omicron variant exhibits a substantial presence of long sequence fragments with perfect matches, while such sequences are almost absent in the Alpha and Delta variants.

In the first scenario, the sequence number of the newly generated 3’ end resulting from the left deletion is noticeably smaller than that of the new 5’ end on the right, indicating the occurrence of a deletion event. In the second situation, the sequence has undergone elongation, which is another crucial factor contributing to the formation of defective viruses, known as copy-backs. To determine typical defective viruses, we established a threshold of 5 nucleotides (nt), considering deletions or insertions of duplicated segments exceeding this length. The two-dimensional site map of defective viruses is visually presented in **Figure 3B**, allowing for the identification of specific sites where deletions or replication duplications occur. The probability of deletions or erroneous insertions at each site is illustrated in **Figure 3C**. By utilizing **Figure 3C**, we can calculate the average occurrence of insertions or deletions across the entire genome. Our analysis involved 297 SARS-CoV-2 second-generation sequencing samples, encompassing 105 Omicron, 93 Delta, and 99 Alpha strains, with the outcomes displayed in **Figure 3D**. **Figure 3D** represents the average probability of each nucleotide position experiencing a defective-type mutation. These mutations extend beyond point mutations and may involve breakages or copy-back events. Remarkably, **Figure 3D** demonstrates that all strains have the potential to generate defective viruses, and the occurrence of defective virus mutations in the Omicron strain did not exhibit a significant decrease. Although the proportion of intact genomes in the Omicron strain is notably higher than that in the Delta and Alpha strains, as observed in **Figure 4C** (later in the study), the proportion of defective viruses is predominantly associated with the time of infection rather than the specific infecting strain.

### 3.3 The mechanisms of defective virus generation revealed by third-generation sequencing data lie in the replication errors of RNA polymerases

At present, a consensus regarding the etiology of defective virus generation remains elusive. While a widely embraced notion implicates replication errors within RNA polymerases, an alternative hypothesis posits the involvement of viral RNA cleavage by host RNAases and recombination facilitated by RNA ligases. Here, we leveraged third-generation sequencing data to explore the plausibility of this alternative mechanism, ultimately demonstrating its non-existence or extreme rarity. Notably, had the second mechanism been operational, the resultant recombinant viral genome would inevitably harbor fragments of host RNA, owing to potential ligation of host transcript fragments during the RNA ligation process. To commence, we employed bioinformatics methodologies to filter out RNA sequences that lack concordance with the viral reference genome in both positive and negative strands, as well as their reverse complements. Subsequently, these sequences could only have originated from exogenous RNA or accumulated replication errors, with the detailed sequences provided in the **Supplementary Materials**. Upon comparative analysis with the human genome and transcriptome, we discerned no substantiating evidence indicating the origin of any RNA fragment from the human transcriptome. Consequently, we refute the second mechanism and attribute defective virus generation to replication errors within RNA polymerases. Our analysis involved 1128 SARS-CoV-2 third-generation sequencing samples (SMRT data), encompassing 384 Omicron (**Supplementary Table S7**), 290 Delta (**Supplementary Table S6**), and 454 Alpha strains (**Supplementary Table S5**). By conducting a comparative analysis of the Alpha, Delta, and Omicron genomic data, intriguing findings emerged. Specifically, when examining the Alpha and Delta strains, the third-generation sequencing data revealed a scarcity of sequences that exhibited complete concordance with the reference genome. In contrast, a substantial proportion of sequences obtained from the Omicron strain demonstrated full matches with the reference genome. Notably, for the alpha and delta variants, the non-matching regions within long sequences were predominantly situated at the ends, whereas the middle regions displayed a high degree of similarity. This phenomenon does not stem from an exceptionally high fidelity of the RNA polymerase in the omicron strain but rather reflects two pivotal mechanisms at play.

Firstly, the replication process of SARS-CoV-2’s RNA polymerase manifests an exceedingly high error rate, surpassing our prior understanding. During the initial stages of replication, a template sequence with a pronounced mismatch rate is synthesized. Subsequently, proteins such as Nsp employ proofreading capabilities to rectify these erroneous bases. The utilization of third-generation sequencing allows for the effective capture of these details, revealing both long and short sequences. Throughout the reverse transcription process, these RNA polymerases are shed, leading to significant base mutations at both ends of the DNA sequence. However, the middle region tends to exhibit a higher degree of concordance. **Figure 4A** illustrates this mechanism.

The second mechanism revolves around the packaging capacity of the Omicron strain, which significantly surpasses that of the Delta and Alpha strains. Consequently, the number of viral particles is substantially greater in the Omicron variant. RNA encapsulated by the capsid proteins often consists of relatively intact RNA sequences with intact 5’ and 3’ structures, particularly after replication completion. These RNA sequences do not harbor RNA polymerases. Thus, we observed a considerable number of long sequence segments from the omicron strain that exhibited full concordance with the reference genome. These fully matching long sequences originate from RNA sequences enclosed within capsid proteins.

However, for the Alpha and Delta strains, due to their significantly lower assembly capabilities compared to the Omicron strain, complete chains are prone to degradation. Consequently, the proportion of intact RNA fragments within the third-generation sequencing data that align with the reference sequence is exceedingly low, rendering it challenging to detect long sequences that exhibit complete concordance. Moreover, for the Omicron strain, a notable number of long sequences with end mutations were also observed, indicating that the reliability of RNA polymerase replication has not undergone significant changes compared to previous strains. **Figure 4B** visually represents this phenomenon, wherein all mutant strains showcase a substantial proportion of long sequences with end mutations.

Although the Ct values of the Omicron variant in nucleic acid detection (quantitative PCR) might slightly exceed those of other strains such as Delta, its genome integrity is markedly higher. This observation can also account for the exceptional infectivity exhibited by the omicron variant. **Figure 4C** presents the proportions of sequences within the third-generation sequencing data that fully match the reference genome for different strains. Notably, **Figure 4C** demonstrates that the Omicron strain encompasses a considerable number of complete genome sequences, surpassing those observed in early strains like Delta and Alpha.

### 3.4 The impact of defective viruses on individual infection

While experimental studies have already confirmed the disruptive role of defective viruses in viral replication, there is currently a lack of systematic research on the impact of defective viruses on individual infection. The presence of defective viruses plays a crucial role in viral evolution and clearance within the host organism.

#### 3.4.1 Defective virus can prevent severe infections

Conventional investigations into host-virus interactions predominantly emphasize the host’s immune function. Nevertheless, it is crucial to acknowledge that viral replication errors resulting in the generation of numerous defective viruses also play a significant role in their eventual eradication by the immune system. In the absence of replication errors induced by viral polymerases, infected individuals may elicit more potent immune responses and present with more severe clinical symptoms.

**Figure 5:**
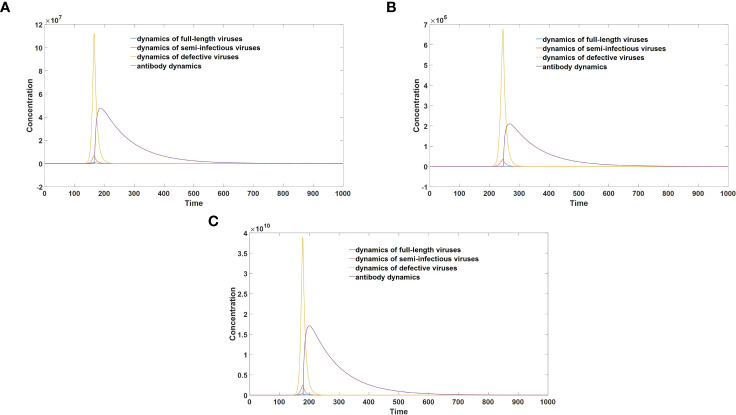
**(A)** Virus-antibody dynamics after semi-infectious virus infection. **(B)** Virus-antibody dynamics after full-length virus infection (replication error = 10%). **(C)** Virus-antibody dynamics after full-length virus infection (replication error = 5%).

The graphical representation in **Figure 5** clearly demonstrates that infections caused by semi-infectious viruses exhibit markedly reduced severity compared to those induced by full-length viruses. Furthermore, a comparative analysis between **Figures 5B, C** elucidates the pivotal role of replication errors in mitigating infection severity. Notably, as the rate of replication errors diminishes, leading to a decreased likelihood of generating defective viruses during each replication cycle, both the peak viral load and peak antibody concentration experience a substantial increase. Consequently, this heightened viral load and antibody response correlate with the manifestation of more severe clinical symptoms.

#### 3.4.2 Generation of defective viruses can reduce the infectivity of individuals in the late stage of infection

**Figure 6:**
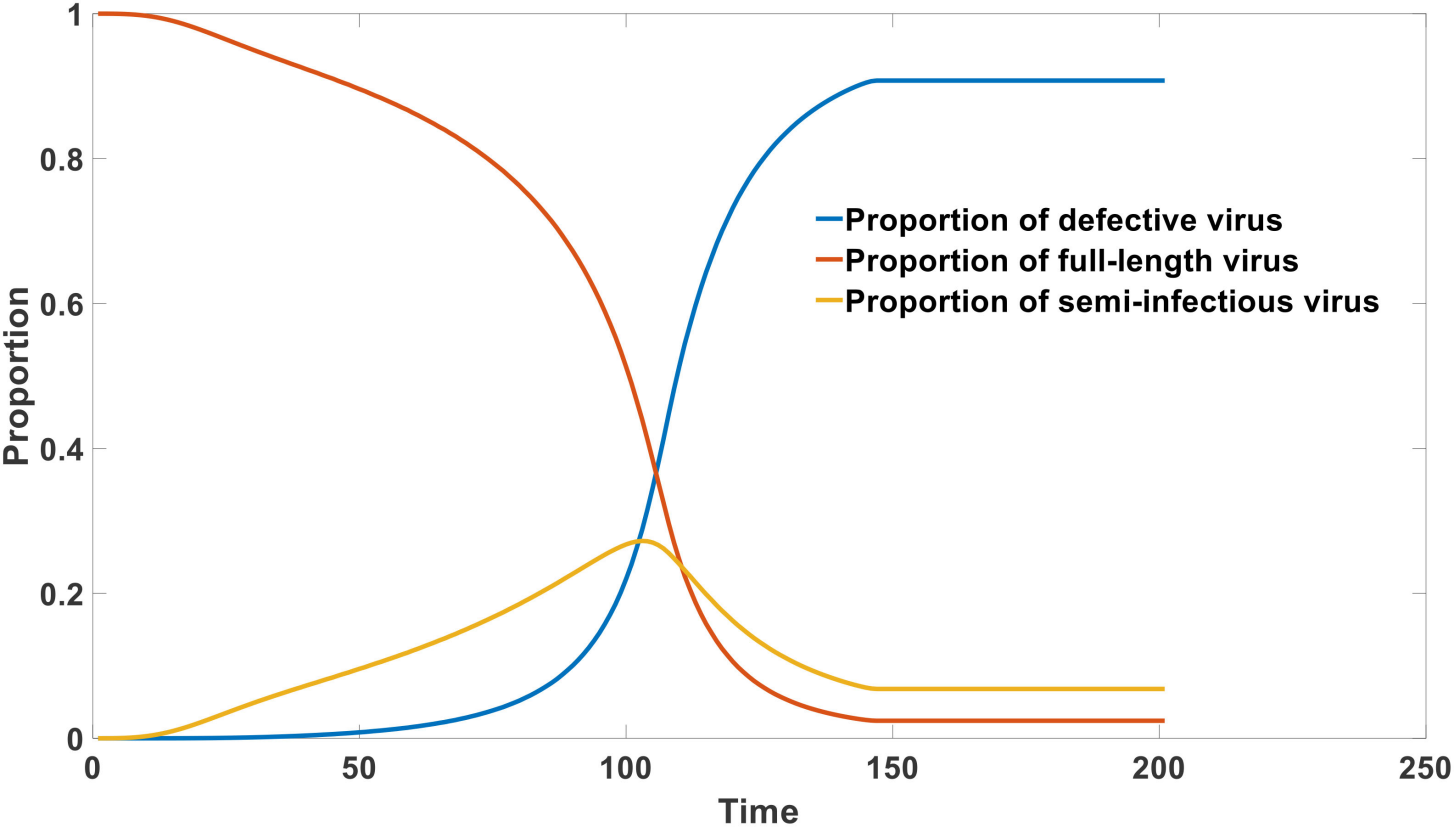
Dynamics of virus composition after infection.

As reported in experimental studies, the proportion of defective viruses significantly increases with the progression of the infection cycle (26). Simultaneously, there is a significant decrease in the proportion and absolute concentration of intact RNA viruses. This phenomenon explains why patients exhibit strong infectivity during the incubation period and early stages of infection, while their infectivity rapidly declines in the later stage of infection. This can be depicted in **Figure 6**.

#### 3.4.3 The host antibody level exerts selective pressure on intact viruses, screening out full-length viruses to ensure that the virus does not succumb to rapid decay caused by replication errors

**Figure 7:**
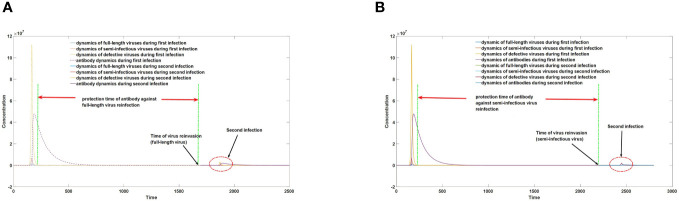
**(A)** protection time against reinfection with full-length virus. **(B)** protection time against reinfection with semi-infectious virus.

Disregarding the interplay between host and virus, a simplistic expectation would be for viruses to rapidly evolve towards lower virulence. For instance, in a hypothetical scenario with a probability *p* of replication errors and generation of defective viruses during each replication cycle, after *N* iterations the proportion of intact viruses would diminish to (*1-p*)*^N^*. Despite the minute magnitude of *p*, as *N* becomes sufficiently large, the proportion of intact viruses would gradually dwindle until their eventual extinction. However, the actual process of viral evolution is more intricate. Consider respiratory viruses like SARS-CoV-2 and influenza virus, which, despite possessing a typical mechanism for generating defective viruses, maintain stable virulence for a prolonged period due to the host’s selective pressure against highly virulent strains. This seemingly paradoxical phenomenon stems from the significant screening effect of antibodies on highly virulent intact viruses. In the absence or presence of extremely low antibody levels, all self-replicating viral particles, including semi-infectious viruses, can invade and proliferate within the host. However, when the host’s antibody level surpasses a certain threshold, weakly replicating genomic segments of defective viruses fail to effectively proliferate. Conversely, insufficient antibody levels fail to completely inhibit the replication of highly replicating intact viruses, enabling their effective proliferation. Following initial infection, hosts often experience a marked increase in antibody levels, which subsequently decline over time. Initially decreasing to a level that permits the replication of intact viral particles, antibody levels then decline to a threshold that facilitates the replication of defective viruses. Through this mechanism, hosts tend to preferentially select intact, highly active viral particles during secondary infection rather than those with defects, as depicted in **Figure 7**. It is important to note that not all viruses undergo such a process. When the value of *p* is substantial, the population’s evolution may still irreversibly progress rapidly towards loss of virulence. Previous research (31) has suggested that the rapid disappearance of SARS and MERS may be attributed to this phenomenon, as truncation of the two UTRs is a common means of generating defective viruses with weak replication activity.

As illustrated in **Figure 7**, augmented levels of antibodies following primary infection provide varying degrees of protection against different viruses. The duration of immunity against semi-infectious viruses greatly surpasses that of full-length viruses. Consequently, a phenomenon arises where the host is vulnerable to full-length viruses but resistant to semi-infectious viruses during the interval between viral invasion at 7A and the invasion of semi-infectious viruses at 7B. This selection pressure favors the survival and evolution of full-length viruses.

### 3.5 How defective viruses impact population infection: a study on the infectious characteristics

Extensive research has been conducted on defective viruses, utilizing both experimental and bioinformatics approaches. However, there remains a notable gap in our understanding of how defective viruses impact the infection dynamics within host populations. The presence of defective viruses may exert significant effects on the infection characteristics of populations, thereby challenging conventional paradigms related to the transmission of infectious diseases. Indeed, numerous infection characteristics within populations are intricately linked to the existence of defective viruses.

For example, environmental factors such as low temperature and dry climate have been observed to favor virus transmission, with many respiratory infections exhibiting major outbreaks during the winter season. Furthermore, these environmental conditions often correspond to more severe infection symptoms. Notably, during the late 2022 Omicron outbreak, patients in northern China demonstrated more severe symptoms compared to those in southern regions, where asymptomatic or mild cases were prevalent. Additionally, the implementation of protective measures significantly influences the symptoms experienced by infected individuals. Strict control measures at the societal level, as well as stringent individual protective measures, not only reduce the likelihood of infection but also diminish the severity of infection. These commonly observed phenomena are closely associated with the presence of defective viruses.

Before employing mathematical models to systematically simulate these processes, it is pertinent to provide a macro-level explanation. Both experimental and theoretical studies have indicated that the peak viral load following infection exhibits little correlation with the initial viral dosage. Rather, it is determined by the virus’s replicative capacity and the host’s immune response. This suggests that protective measures primarily reduce the probability of infection rather than the severity of infection. However, for SARS-CoV-2 infections, empirical evidence suggests that reducing the initial viral dosage can mitigate the symptoms experienced by infected individuals, as evidenced in statistical studies on the effectiveness of mask protection. This phenomenon can be attributed to the heterogeneity of viral infections, wherein significant genetic variations exist within the same strain of the novel coronavirus.

To expound upon this, consider the scenario where the proportion of intact viral particles within the total viral population is 1%. When a host inhales the virus, they are essentially inhaling a proportion of intact viral particles equal to 1%. However, with each subsequent inhalation, the proportion of intact viral particles decreases. Additionally, the presence of semi-infectious particles and non-functional defective viruses further complicates the viral landscape. Non-functional defective viruses, lacking autonomous replicative ability, do not cause infections. Conversely, mixed infections involving defective viruses often result in an overall decrease in viral replicative capacity, leading to milder clinical symptoms. Defective viruses with partial functionality exhibit weaker replicative abilities compared to normal viruses, resulting in lower peak viral loads and weaker antibody responses, thereby yielding less severe infection symptoms. In contrast, complete viruses capable of infecting individuals typically cause severe infections. Therefore, infections involving semi-infectious particles manifest milder symptoms and remain transmissible, subsequently leading to less severe clinical symptoms in subsequent infections. Mathematical models offer a comprehensive means to simulate and elucidate this macro-level phenomenon.

**Figure 8:**
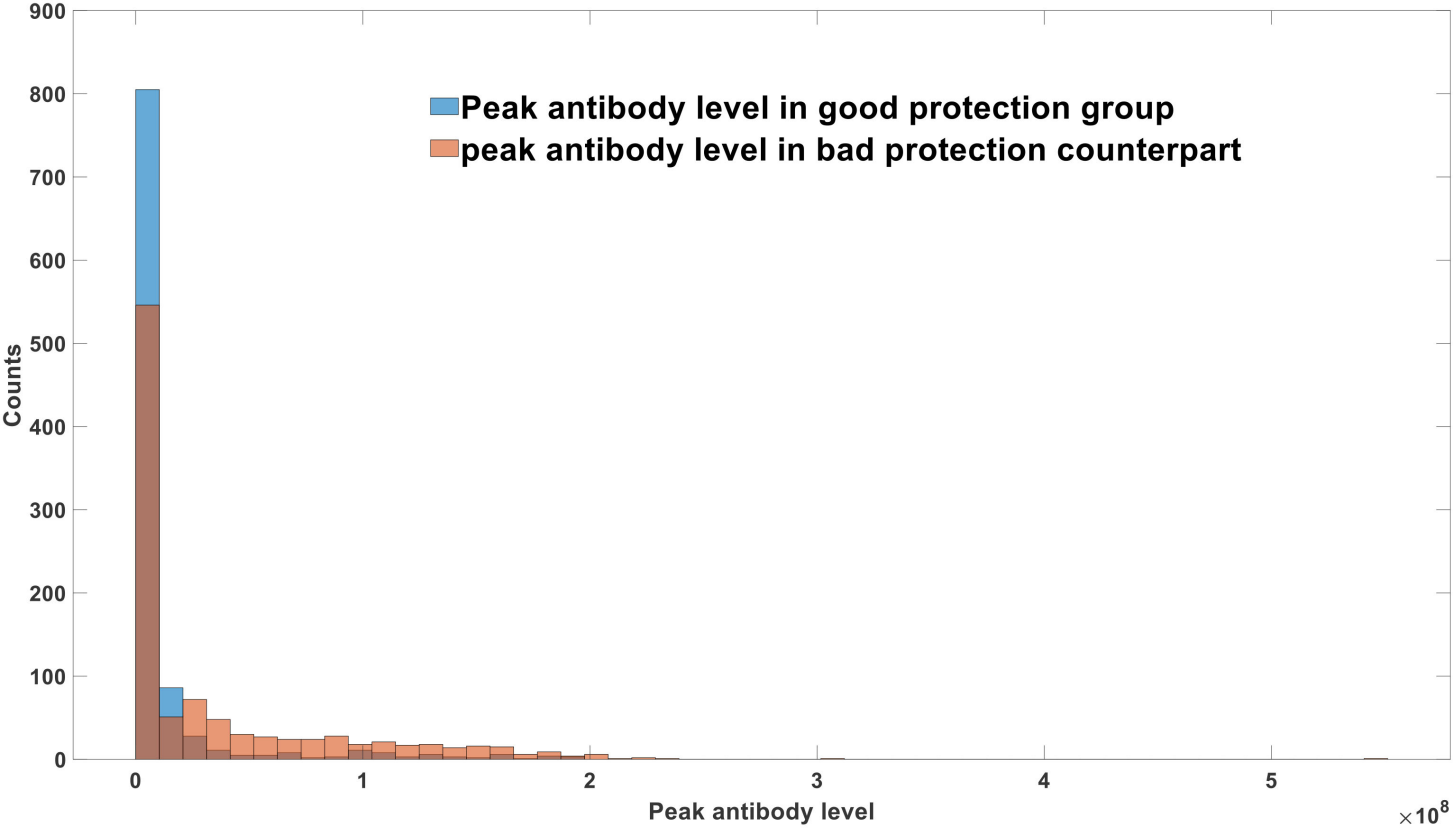
Effects of protection measure on the infection severity at population level.

By adjusting the parameter representing the number of environmental viruses inhaled by each individual in the population, we can modulate different protective measures as well as the influence of environmental conditions and temperature on infection dynamics. Higher values of this parameter signify lower control measures or colder environments, resulting in an increased number of environmental viruses inhaled by individuals. Conversely, lower values of the parameter denote strict control measures or hot and humid external environments. **Figure 8** demonstrates that effective control measures and humid climate conditions substantially reduce peak antibody levels and viral loads in the population, correlating with milder infection symptoms.

## 4 Discussion

Our research on defective viruses aims to elucidate many perplexing phenomena observed in COVID-19 patients, such as the significant variability in symptom presentation among individuals infected with the same viral strain. This variation in symptoms cannot always be attributed solely to differences in individual immune responses, suggesting the presence of viral heterogeneity within the environment. Additionally, the diversity in population infection characteristics can also be better explained by the existence of Defective Viral Genomes (DVGs).

An illustrative example of this is evident in the contradictions and confusion observed among infectious disease experts during the COVID-19 outbreak in China, particularly following the emergence of the Omicron strain. Mortality data from different regions exhibited notable deviations, even when considering the same viral subtype. For instance, tropical areas like Singapore demonstrated lower death rates compared to developed countries such as North America. In early December 2022, China lifted its epidemic prevention policy, and prior to that, the mortality rate caused by the Omicron strain in China was very low. However, upon lifting the policy, there was a substantial increase in deaths attributed to Omicron in China, estimated conservatively at over 5 million. Importantly, individuals infected in northern China exhibited predominantly high fever symptoms, unlike before when asymptomatic infections were more prevalent. Conversely, southern China experienced a less severe situation, with a significantly higher proportion of asymptomatic infections compared to the north (9, 10).

To demonstrate the prevalence of defective viral genomes (DVGs) in SARS-CoV-2 infections, we employed bioinformatics techniques for DVG mining and statistical analysis. Our methodology was largely based on the definitions and research approaches set forth by researchers Carolina B. López (18, 23) and Yan Sun (26). We categorized DVGs into two major types: deletions and copybacks. DVG mining was achieved through the alignment of sequencing fragments to a reference genome. However, unlike the study by researcher Sun (26), we explicitly investigated the sites generated by copyback events.

While the previous research focused primarily on examining the changing proportions of DVGs in different patients over time (26, 31), our study aimed to compare the genomic homogeneity of SARS-CoV-2 with that of other DNA viruses, thereby highlighting the more pronounced occurrence of DVGs in SARS-CoV-2. Furthermore, we compared the differences in DVGs among different SARS-CoV-2 strains. Based on second-generation sequencing data, there appeared to be no significant variation in the proportion of defective viruses among different strains. The changes in this proportion were solely associated with the infection cycle, meaning that it increased as the infection progressed. This finding is consistent with the reports by researcher B.

In a departure from previous studies, we further conducted DVG analysis on different SARS-CoV-2 strains using long sequence fragments obtained through SMRT sequencing. This yielded meaningful and astonishing results. Firstly, we discovered a large number of sequencing fragments with mismatches at both ends compared to the reference genome. This suggests a possible replication mechanism of the RNA polymerase, wherein rapid replication of the template strand is achieved through a low fidelity but highly efficient process, followed by subsequent error correction and replacement by error-correcting enzymes. Only this mechanism can account for the abundant end-mismatched sequences observed in the SMRT data. Additionally, we found that nearly only the omicron strain exhibited a considerable proportion of long sequence fragments that matched the reference genome perfectly. This indicates the superior assembly capability of the omicron strain compared to the delta and alpha strains. Theoretical predictions (29) and microscopic experiments (32–34) have corroborated this finding from different perspectives. Moreover, the presence of intact omicron viral particles in the samples explains why omicron exhibits exceptionally high transmissibility.

After the ubiquity of defective viral genomes (DVGs) in SARS-CoV-2 has been demonstrated, we aim to further establish a connection between the genetic attributes of the virus and the infective properties of the population through mathematical modeling. To our knowledge, this is an area that previous simulation studies have not ventured into, thus lacking comparative discussions with prior work. By developing a mathematical model, we were able to quantitatively evaluate the influence of viral heterogeneity on individual susceptibility during infection. Our model offers insights into the advantageous role of defective viruses in eliciting host immune responses, elucidates the progressive increase in their proportions throughout the course of infection, and correlates with alterations in the transmissibility of infected individuals. Moreover, our model provides clarification on how the virus can sustain its original genotype or length during evolution without rapid deterioration into low-virulence or non-virulent defective viruses.

Population infection models built upon mathematical frameworks allowed us to further examine the influence of climate conditions and social control measures on disease manifestation within populations. Our model highlights the strong correlation between individual infection characteristics and the viral inoculum dose. Specifically, colder climate conditions and less stringent control measures can exacerbate the severity of symptoms within populations, thereby providing an explanation for the pronounced regional disparities in mortality rates observed during the course of SARS-CoV-2 infection.

Therefore, when you feel sick, do not be overly disappointed with your immune system, as you may be infected with a highly virulent non-defective virus. Similarly, when you become an asymptomatic carrier, do not assume that your immune system is exceptionally robust, as you may simply be infected with a defective virus.

However, we must acknowledge the limitations of this mathematical model. Firstly, for simplicity, our model compartmentalizes the virus into discrete modules or compartments, whereas in reality, the distribution of defective viruses is continuous, and the population of DVGs may encompass a vast array of distinct sequences, potentially numbering in the millions. Thus, this model greatly simplifies the actual dynamics of population infection. Additionally, our model lacks a quantitative data fitting process, and the selection of model parameters is merely aimed at maximizing the explanation and satisfaction of macroscopic phenomena. This is largely constrained by the limited availability of experimental data on DVGs. Currently, it is challenging to find clinical data that includes the quantities of different DVGs at various stages of infection and information on antibodies within patients. Furthermore, capturing antibody information often relies on arbitrary units since researchers are primarily interested in relative changes in IgG concentration rather than absolute concentrations. While it is theoretically feasible to determine the replication cycle and protein translation rate of a specific DVG through *in vitro* experiments, the practical significance is not evident due to the immense diversity of SARS-CoV-2 DVGs and their potential interconversions.

While the COVID-19 epidemic is nearly over, the possibility of future similar infectious diseases remains high. The aim of our research is not merely to emphasize the necessity of prevention and control policies, but to uncover the decay and transmission mechanisms of RNA viruses. Perhaps the most valuable application of this research lies in inspiring future vaccine development. Although we have not specifically identified the nucleic acid sequence of this semi-infectious SARS-CoV-2, we have macroscopically demonstrated that this semi-infectious particle can cause a large number of asymptomatic infections in population infection scenarios. Therefore, future vaccine development should consider utilizing these weakly pathogenic virus particles. The concept of using self-replicating live viruses as vaccines has been proposed and practiced by various researchers, such as the group led by Peiyong Shi (35), which employed a partially genome-deleted virus as a vaccine.

## 5 Conclusion

This study investigated the presence and characteristics of DVGs in SARS-CoV-2 infections through analysis of high-throughput sequencing data. The results revealed widespread occurrence of DVGs in the SARS-CoV-2 genome. Comparative analysis with diverse DNA viruses highlighted the notable genome heterogeneity and a significant proportion of recombination sites in SARS-CoV-2. Furthermore, the investigation explored the differences in sequencing depth and DVGs content among various strains, with the Omicron strain showing a higher proportion of intact genomes than the Delta and Alpha strains. This finding suggests that the increased infectiousness of the Omicron strain may be attributed to enhanced virus assembly capacity, reducing the exposure time of vulnerable virus RNA and mitigating its degradation.

Additionally, mathematical modeling simulations provided insights into the impact of DVG effects on infection characteristics and population evolution under varying environmental factors. Specifically, the presence of DVGs significantly reduces the severity of infection. Moreover, the proportion of DVGs increases with the duration of infection, which can reduce the infectivity of individuals in the late stage of infection. Our mathematical model also explains the role of DVGs in virus evolution and why viruses do not degrade completely towards DVGs but are able to maintain the original genome length. Finally, our model explains why physical protective measures and climate conditions can cause significant differences in population infection characteristics for the same viral strain. These findings offer a novel approach for future virus research and vaccine development.

## Data availability statement

The original contributions presented in the study are included in the article/**Supplementary Material**. Further inquiries can be directed to the corresponding author.

## Author contributions

ZX: Conceptualization, Funding acquisition, Methodology, Writing – original draft. QP: Data curation, Writing – original draft. JS: Methodology, Writing – original draft. HZ: Investigation, Validation, Writing – original draft. DW: Writing – review & editing. JD: Writing – review & editing. QZ: Funding acquisition, Supervision, Writing – review & editing.
