# Supplementary material for: Bioinformatic analysis of defective viral genomes in SARS-CoV-2 and its impact on population infection characteristics
Source: Front Immunol. 2024 Jan 29;15:1341906. doi: 10.3389/fimmu.2024.1341906 (PMC10859446; doi:10.3389/fimmu.2024.1341906)
Supplement: Supplementary file 1 [file Table_1.docx]

| Sequence Accession ID | Species | SRA Accession ID | Genome Length (bp) |
| --- | --- | --- | --- |
| NC_071143 | *Flyfo siphovirus Tbat1_6* | SRR16952314 | 48082 |
| NC_070875 | *Serratia phage Scapp* | SRR8788475 | 42969 |
| NC_069741 | *Pseudomonas phage Kaya* | SRR16248205 | 43067 |
| NC_069743 | *Pseudomonas phage Guyu* | SRR16248204 | 43141 |
| NC_069744 | *Xanthomonas phage Samson* | SRR8892199 | 43314 |
| NC_069746 | *Pseudomonas phage Kopi* | SRR16248203 | 42820 |
| NC_069748 | *Pseudomonas phage TehO* | SRR16248202 | 43015 |
| NC_069149 | *Salmonella phage vB_SenTO17* | SRR12933168 | 41658 |
| NC_069152 | *Salmonella phage Shemara* | SRR8869235 | 44342 |
| NC_055814 | *Staphylococcus phage Portland* | SRR8761742 | 17711 |

Table S.1 list of DNA viruses

| Sequence Accession ID | SRA Accession ID |
| --- | --- |
| OL533307 | SRR19014853 |
| OL543608 | SRR16922060 |
| OL514264 | SRR16965765 |
| OL366847 | SRR16790676 |
| OL367846 | SRR16677439 |
| OK653053 | SRR16585809 |
| OK623324 | SRR16563979 |
| OK541433 | SRR16103853 |
| OK547415 | SRR16174488 |
| OK528128 | SRR16348243 |
| OK507849 | SRR16319056 |
| OK421903 | SRR16220602 |
| OK383515 | SRR16145237 |
| OK331678 | SRR16099008 |
| OK289724 | SRR16069731 |
| OK289731 | SRR16070120 |
| MZ933887 | SRR15651766 |
| MZ916262 | SRR19493370 |
| MZ908066 | SRR19461361 |
| MZ911531 | SRR19928767 |
| MZ842889 | SRR15512968 |
| MZ832693 | SRR15505294 |
| MZ817669 | SRR15435494 |
| MZ801287 | SRR15463089 |
| MZ772098 | SRR15404775 |
| MZ750305 | SRR15390578 |
| MZ734506 | SRR15342039 |
| MZ727189 | SRR15306226 |
| MZ733886 | SRR15296700 |
| MZ703731 | SRR15224781 |
| MZ703829 | SRR15224235 |
| MZ704129 | SRR15224982 |
| MZ704548 | SRR15138677 |
| MZ704657 | SRR15139057 |
| MZ704866 | SRR15138298 |
| MZ705236 | SRR15139172 |
| MZ706239 | SRR15200885 |
| MZ670011 | SRR15162905 |
| MZ672009 | SRR15295103 |
| MZ648346 | SRR15238799 |
| MZ662219 | SRR15239114 |
| MZ615549 | SRR15209106 |
| MZ578276 | SRR15196433 |
| MZ592806 | SRR15139351 |
| MZ574782 | SRR15188648 |
| MZ557505 | SRR15096485 |
| MZ520932 | SRR15064801 |
| MZ508335 | SRR15054263 |
| MZ489814 | SRR14653932 |
| MZ491226 | SRR14999903 |
| MZ494045 | SRR14999839 |
| MZ470749 | SRR14658194 |
| MZ472513 | SRR14997896 |
| MZ476093 | SRR14919087 |
| MZ467041 | SRR14919038 |
| MZ444206 | SRR14882506 |
| MZ377321 | SRR14790695 |
| MZ256516 | SRR14610032 |
| MZ256553 | SRR14610011 |
| MZ267394 | SRR14241235 |
| MZ256516 | SRR14610032 |
| MZ256797 | SRR14609310 |
| MZ267761 | SRR14626095 |
| MZ207507 | SRR14555132 |
| MZ194685 | SRR14546538 |
| MZ202901 | SRR14447187 |
| MZ149469 | SRR14474428 |
| MZ149623 | SRR14474771 |
| MZ145400 | SRR14463812 |
| MZ101863 | SRR14428757 |
| MZ068834 | SRR14378285 |
| MZ068890 | SRR14378844 |
| MZ032331 | SRR14330920 |
| MZ032351 | SRR14331042 |
| MW915171 | SRR14225645 |
| MW915226 | SRR14225386 |
| MW922026 | SRR14208879 |
| MW921976 | SRR14208606 |
| MW793618 | SRR14028169 |
| MW796736 | SRR13958139 |
| MW788796 | SRR13924579 |
| MW788871 | SRR13924612 |
| MW767573 | SRR13980430 |
| MW767584 | SRR13980418 |
| MW750908 | SRR13957128 |
| MW750901 | SRR13957126 |
| MW741604 | SRR13919919 |
| MW750132 | SRR13971633 |
| MW727236 | SRR13928111 |
| MW715154 | SRR13867885 |
| MW715228 | SRR13868020 |
| MW715292 | SRR13867970 |
| MW692971 | SRR13834191 |
| MW687499 | SRR13835776 |
| MW684541 | SRR13808723 |
| MW684706 | SRR13808733 |
| MW660973 | SRR13765900 |
| MW648279 | SRR13748712 |
| MW645990 | SRR13502267 |

Table S.2 list of Alpha strains with NGS data (second generation sequence data)

| Sequence Accession ID | SRA Accession ID |
| --- | --- |
| ON686049 | SRR19522043 |
| ON618702 | SRR19205633 |
| ON618839 | SRR19206006 |
| ON618841 | SRR19206004 |
| ON618961 | SRR19205849 |
| ON619264 | SRR19389319 |
| ON619269 | SRR19389314 |
| ON563159 | SRR19359256 |
| ON513732 | SRR19477535 |
| ON472248 | SRR19471193 |
| ON472546 | SRR19468281 |
| ON472736 | SRR19468274 |
| ON472756 | SRR19468158 |
| ON473302 | SRR19468571 |
| ON473585 | SRR19467243 |
| ON474074 | SRR19468706 |
| ON474119 | SRR19468792 |
| ON474586 | SRR19473753 |
| ON421811 | SRR19471637 |
| ON226081 | SRR18728295 |
| ON214164 | SRR18722265 |
| ON185752 | SRR18642882 |
| ON185753 | SRR18642885 |
| ON186017 | SRR18644882 |
| ON186056 | SRR18644828 |
| ON186058 | SRR18644851 |
| ON186062 | SRR18644843 |
| ON187878 | SRR18643121 |
| ON113790 | SRR18518759 |
| ON094481 | SRR18504278 |
| OM994864 | SRR18278580 |
| OM702547 | SRR18027265 |
| OM689003 | SRR18026627 |
| OM689064 | SRR18026608 |
| OM689127 | SRR18026641 |
| OM690826 | SRR18025839 |
| OM690879 | SRR18025724 |
| OM690880 | SRR18025723 |
| OM690881 | SRR18025722 |
| OM690882 | SRR18025721 |
| OM690886 | SRR18025715 |
| OM691218 | SRR18026124 |
| OM663346 | SRR18002316 |
| OM648465 | SRR17958626 |
| OM639820 | SRR17958626 |
| OM643007 | SRR17960205 |
| OM524823 | SRR17879234 |
| OM524828 | SRR17879229 |
| OM524833 | SRR17878968 |
| OM524868 | SRR17878759 |
| OM524909 | SRR17878953 |
| OM524924 | SRR17878936 |
| OM525009 | SRR17878792 |
| OM525404 | SRR17879456 |
| OM476006 | SRR18797306 |
| OM487179 | SRR17860830 |
| OM487343 | SRR17860496 |
| OM455894 | SRR20504103 |
| OM458310 | SRR17836223 |
| OM458757 | SRR17836206 |
| OM437280 | SRR17815439 |
| OM432676 | SRR17628148 |
| OM433438 | SRR17626188 |
| OM433525 | SRR17588060 |
| OM433559 | SRR17588195 |
| OM433607 | SRR17588141 |
| OM433679 | SRR17587989 |
| OM433680 | SRR17587988 |
| OM433681 | SRR17587987 |
| OM433682 | SRR17587986 |
| OM390365 | SRR17751036 |
| OM390484 | SRR17751016 |
| OM390614 | SRR17753372 |
| OM393192 | SRR21089412 |
| OM400856 | SRR17432629 |
| OM400942 | SRR17432505 |
| OM349734 | SRR19939717 |
| OM352376 | SRR17701222 |
| OM346365 | SRR17707453 |
| OM335726 | SRR20881443 |
| OM309513 | SRR20857043 |
| OM294291 | SRR17648201 |
| OM294579 | SRR17633680 |
| OM294866 | SRR17647155 |
| OM294931 | SRR17646890 |
| OM267281 | SRR17616954 |
| OM191603 | SRR17510167 |
| OM202035 | SRR20508032 |
| OM202070 | SRR20508126 |
| OM202779 | SRR20531521 |
| OM173291 | SRR19322924 |
| OM182468 | SRR17487341 |
| OM182512 | SRR17487343 |

Table S.3 list of Delta strains with NGS data (second generation sequence data)

| Sequence Accession ID | SRA Accession ID |
| --- | --- |
| OQ458224 | SRR23497515 |
| OQ458243 | SRR23497541 |
| OQ458421 | SRR23497474 |
| OQ452291 | SRR23505101 |
| OQ452545 | SRR23505297 |
| OQ444536 | SRR23494633 |
| OQ441367 | SRR23462198 |
| OQ441753 | SRR23462232 |
| OQ431135 | SRR23453252 |
| OQ431157 | SRR23453499 |
| OQ431184 | SRR23453240 |
| OQ431267 | SRR23453242 |
| OQ431378 | SRR23453490 |
| OQ431828 | SRR23452408 |
| OQ432276 | SRR23452416 |
| OQ433225 | SRR23451198 |
| OQ433389 | SRR23451497 |
| OQ425562 | SRR23411169 |
| OQ426317 | SRR23411031 |
| OQ422383 | SRR23424954 |
| OQ419481 | SRR23391296 |
| OQ419482 | SRR23391297 |
| OQ419648 | SRR23391050 |
| OQ419686 | SRR23391051 |
| OQ419690 | SRR23391049 |
| OQ419943 | SRR23391571 |
| OQ421293 | SRR23404123 |
| OQ421298 | SRR23404161 |
| OQ421355 | SRR23404149 |
| OQ408000 | SRR23373057 |
| OQ408366 | SRR23373532 |
| OQ399579 | SRR23359160 |
| OQ400638 | SRR23359254 |
| OQ381422 | SRR23338951 |
| OQ382020 | SRR23342790 |
| OQ382143 | SRR23342482 |
| OQ382145 | SRR23342914 |
| OQ380248 | SRR23339480 |
| OQ368748 | SRR23305998 |
| OQ368750 | SRR23305991 |
| OQ368751 | SRR23305996 |
| OQ368752 | SRR23306029 |
| OQ368769 | SRR23306035 |
| OQ368772 | SRR23306036 |
| OQ368792 | SRR23305997 |
| OQ368799 | SRR23305992 |
| OQ368802 | SRR23305969 |
| OQ368814 | SRR23305980 |
| OQ368826 | SRR23305985 |
| OQ368842 | SRR23305151 |
| OQ368872 | SRR23305194 |
| OQ368873 | SRR23305193 |
| OQ368888 | SRR23305142 |
| OQ368960 | SRR23305525 |
| OQ369250 | SRR23315619 |
| OQ369610 | SRR23316122 |
| OQ371489 | SRR23319261 |
| OQ371526 | SRR23319279 |
| OQ371527 | SRR23319205 |
| OQ371540 | SRR23319220 |
| OQ371546 | SRR23319215 |
| OQ371570 | SRR23319130 |
| OQ371573 | SRR23319093 |
| OQ371640 | SRR23319070 |
| OQ371668 | SRR23319184 |
| OQ371669 | SRR23319183 |
| OQ371718 | SRR23321975 |
| OQ371719 | SRR23321977 |
| OQ372133 | SRR23322232 |
| OQ372171 | SRR23322228 |
| OQ372174 | SRR23322167 |
| OQ362554 | SRR22831296 |
| OQ362562 | SRR22831281 |
| OQ362587 | SRR22831322 |
| OQ362590 | SRR22831326 |
| OQ362610 | SRR22831260 |
| OQ362620 | SRR22831321 |
| OQ362623 | SRR22831297 |
| OQ362649 | SRR22991898 |
| OQ362664 | SRR22991933 |
| OQ362716 | SRR22991907 |
| OQ362730 | SRR22812642 |
| OQ362756 | SRR22812582 |
| OQ362772 | SRR22812634 |
| OQ362844 | SRR22812527 |
| OQ362869 | SRR22991783 |
| OQ362876 | SRR22991797 |
| OQ362896 | SRR22991745 |
| OQ362908 | SRR22991799 |
| OQ362925 | SRR22991747 |
| OQ362929 | SRR22991786 |
| OQ356660 | SRR23282676 |
| OQ356710 | SRR23283093 |
| OQ347040 | SRR23259277 |
| OQ347098 | SRR23259142 |
| OQ347120 | SRR23259173 |
| OQ352311 | SRR23253710 |
| OQ352319 | SRR23253725 |
| OQ352326 | SRR23253697 |
| OQ352356 | SRR23253670 |
| OQ352404 | SRR23253609 |
| OQ346040 | SRR23264954 |
| OQ344979 | SRR23258606 |
| OQ345091 | SRR23258590 |
| OQ345229 | SRR23258529 |

Table S.4 list of Omicron strains with NGS data (second generation sequence data)

| Sequence Accession ID | SRA Accession ID |
| --- | --- |
| MZ785597 | SRR15382420 |
| MZ785599 | SRR15378520 |
| MZ785608 | SRR15382231 |
| MZ785613 | SRR15382403 |
| MZ785614 | SRR15382399 |
| MZ785615 | SRR15378517 |
| MZ785619 | SRR15382417 |
| MZ591639 | SRR15156521 |
| MZ591653 | SRR15156515 |
| MZ591656 | SRR15156504 |
| MZ591872 | SRR15157358 |
| MZ592195 | SRR15156148 |
| MZ592196 | SRR15156156 |
| MZ592199 | SRR15156157 |
| MZ592201 | SRR15156155 |
| MZ592204 | SRR15156152 |
| MZ592205 | SRR15156158 |
| MZ592213 | SRR15157230 |
| MZ592214 | SRR15156231 |
| MZ592215 | SRR15156229 |
| MZ592219 | SRR15157234 |
| MZ592221 | SRR15156233 |
| MZ592222 | SRR15156145 |
| MZ592232 | SRR15157242 |
| MZ592235 | SRR15157243 |
| MZ592236 | SRR15157238 |
| MZ592246 | SRR15156183 |
| MZ592249 | SRR15156162 |
| MZ592256 | SRR15156202 |
| MZ592257 | SRR15156201 |
| MZ592263 | SRR15156132 |
| MZ592265 | SRR15157235 |
| MZ592269 | SRR15156206 |
| MZ592270 | SRR15156140 |
| MZ592273 | SRR15156168 |
| MZ592275 | SRR15156129 |
| MZ592291 | SRR15156192 |
| MZ592470 | SRR15157258 |
| MZ553939 | SRR15087858 |
| MZ553942 | SRR15087869 |
| MZ553948 | SRR15087746 |
| MZ553950 | SRR15087999 |
| MZ553969 | SRR15087892 |
| MZ553971 | SRR15087890 |
| MZ553973 | SRR15087920 |
| MZ553975 | SRR15087827 |
| MZ553976 | SRR15087919 |
| MZ553981 | SRR15088004 |
| MZ553983 | SRR15087837 |
| MZ553986 | SRR15087777 |
| MZ553991 | SRR15087781 |
| MZ553998 | SRR15087978 |
| MZ554004 | SRR15087949 |
| MZ554006 | SRR15087974 |
| MZ554024 | SRR15087987 |
| MZ554025 | SRR15087939 |
| MZ554027 | SRR15087988 |
| MZ554032 | SRR15087983 |
| MZ554035 | SRR15087955 |
| MZ554036 | SRR15087950 |
| MZ554037 | SRR15087940 |
| MZ554048 | SRR15087899 |
| MZ554050 | SRR15087954 |
| MZ554055 | SRR15087792 |
| MZ554090 | SRR15087942 |
| MZ554163 | SRR15088034 |
| MZ554164 | SRR15088010 |
| MZ554169 | SRR15088026 |
| MZ554180 | SRR15088015 |
| MZ554182 | SRR15088012 |
| MZ554183 | SRR15088055 |
| MZ554190 | SRR15088019 |
| MZ554207 | SRR15088305 |
| MZ554334 | SRR15088388 |
| MZ554339 | SRR15087725 |
| MZ554347 | SRR15088377 |
| MZ554358 | SRR15088378 |
| MZ554381 | SRR15088182 |
| MZ554383 | SRR15088180 |
| MZ554387 | SRR15088187 |
| MZ554388 | SRR15088186 |
| MZ554392 | SRR15088118 |
| MZ554393 | SRR15088179 |
| MZ554419 | SRR15088626 |
| MZ554425 | SRR15088634 |
| MZ554426 | SRR15088628 |
| MZ554431 | SRR15088161 |
| MZ554437 | SRR15088114 |
| MZ554451 | SRR15088174 |
| MZ554465 | SRR15088112 |
| MZ554473 | SRR15088124 |
| MZ554478 | SRR15088122 |
| MZ554493 | SRR15088206 |
| MZ554586 | SRR15088680 |
| MZ554587 | SRR15088681 |
| MZ554664 | SRR15088438 |
| MZ554666 | SRR15088569 |
| MZ554667 | SRR15088577 |
| MZ554669 | SRR15088575 |
| MZ554686 | SRR15089080 |
| MZ554857 | SRR15088826 |
| MZ554861 | SRR15088827 |
| MZ554862 | SRR15088829 |
| MZ554865 | SRR15088734 |
| MZ554867 | SRR15089399 |
| MZ554870 | SRR15088843 |
| MZ554871 | SRR15088842 |
| MZ554874 | SRR15088831 |
| MZ554875 | SRR15088840 |
| MZ554876 | SRR15088850 |
| MZ554877 | SRR15088851 |
| MZ554882 | SRR15088833 |
| MZ554886 | SRR15088805 |
| MZ554889 | SRR15088807 |
| MZ554895 | SRR15089222 |
| MZ554904 | SRR15089350 |
| MZ554905 | SRR15089348 |
| MZ554911 | SRR15089400 |
| MZ554921 | SRR15088813 |
| MZ554922 | SRR15088809 |
| MZ554923 | SRR15088810 |
| MZ554924 | SRR15088801 |
| MZ554933 | SRR15089218 |
| MZ554938 | SRR15089217 |
| MZ554940 | SRR15088837 |
| MZ554944 | SRR15088817 |
| MZ554945 | SRR15088815 |
| MZ554946 | SRR15088877 |
| MZ554953 | SRR15088853 |
| MZ554955 | SRR15088876 |
| MZ554956 | SRR15088875 |
| MZ554959 | SRR15088741 |
| MZ554970 | SRR15088838 |
| MZ554984 | SRR15089213 |
| MZ554985 | SRR15089140 |
| MZ554993 | SRR15088816 |
| MZ554995 | SRR15088745 |
| MZ555002 | SRR15088873 |
| MZ555007 | SRR15088845 |
| MZ555009 | SRR15088844 |
| MZ555010 | SRR15088841 |
| MZ555011 | SRR15089132 |
| MZ555012 | SRR15088865 |
| MZ555015 | SRR15088944 |
| MZ555023 | SRR15089208 |
| MZ555032 | SRR15088753 |
| MZ555035 | SRR15088934 |
| MZ555054 | SRR15089318 |
| MZ555076 | SRR15089122 |
| MZ555090 | SRR15088916 |
| MZ555104 | SRR15089252 |
| MZ555105 | SRR15089248 |
| MZ555107 | SRR15089117 |
| MZ555113 | SRR15089236 |
| MZ555129 | SRR15088761 |
| MZ555130 | SRR15088762 |
| MZ555134 | SRR15088947 |
| MZ555136 | SRR15088764 |
| MZ555139 | SRR15088935 |
| MZ555150 | SRR15088776 |
| MZ555153 | SRR15088778 |
| MZ555161 | SRR15089245 |
| MZ555162 | SRR15089263 |
| MZ555163 | SRR15088917 |
| MZ555169 | SRR15089255 |
| MZ555170 | SRR15089258 |
| MZ555175 | SRR15088767 |
| MZ555176 | SRR15089043 |
| MZ555178 | SRR15089044 |
| MZ555182 | SRR15088482 |
| MZ555187 | SRR15088425 |
| MZ555193 | SRR15088475 |
| MZ555202 | SRR15088478 |
| MZ555211 | SRR15088559 |
| MZ555223 | SRR15088547 |
| MZ555227 | SRR15088556 |
| MZ555230 | SRR15088553 |
| MZ555231 | SRR15088409 |
| MZ555242 | SRR15088533 |
| MZ555248 | SRR15088515 |
| MZ555258 | SRR15089036 |
| MZ555263 | SRR15088525 |
| MZ555273 | SRR15088522 |
| MZ555282 | SRR15088518 |
| MZ555297 | SRR15088396 |
| MZ555298 | SRR15089051 |
| MZ555323 | SRR15089387 |
| MZ555328 | SRR15088511 |
| MZ530843 | SRR15026658 |
| MZ530845 | SRR15026632 |
| MZ530847 | SRR15026592 |
| MZ530848 | SRR15026590 |
| MZ530849 | SRR15026591 |
| MZ530856 | SRR15026026 |
| MZ530857 | SRR15026583 |
| MZ530862 | SRR15026800 |
| MZ530869 | SRR15026574 |
| MZ530870 | SRR15026793 |
| MZ530874 | SRR15027004 |
| MZ530876 | SRR15026573 |
| MZ530879 | SRR15026580 |
| MZ530882 | SRR15026805 |
| MZ530883 | SRR15026411 |
| MZ530885 | SRR15026881 |
| MZ530886 | SRR15027022 |
| MZ530892 | SRR15027006 |
| MZ530900 | SRR15027007 |
| MZ530906 | SRR15026568 |
| MZ530912 | SRR15026430 |
| MZ530918 | SRR15026566 |
| MZ530923 | SRR15026339 |
| MZ530926 | SRR15026797 |
| MZ530928 | SRR15026790 |
| MZ530930 | SRR15026336 |
| MZ530933 | SRR15026418 |
| MZ530934 | SRR15026412 |
| MZ530937 | SRR15027012 |
| MZ530939 | SRR15026582 |
| MZ530946 | SRR15026890 |
| MZ530953 | SRR15026415 |
| MZ530955 | SRR15026806 |
| MZ530956 | SRR15026886 |
| MZ530959 | SRR15026027 |
| MZ530961 | SRR15026786 |
| MZ530962 | SRR15026785 |
| MZ530963 | SRR15026905 |
| MZ530964 | SRR15026882 |
| MZ530965 | SRR15026883 |
| MZ530966 | SRR15026893 |
| MZ530967 | SRR15026903 |
| MZ530969 | SRR15027003 |
| MZ530970 | SRR15026906 |
| MZ530973 | SRR15026784 |
| MZ530976 | SRR15026782 |
| MZ530978 | SRR15026783 |
| MZ530982 | SRR15026640 |
| MZ530987 | SRR15026656 |
| MZ530988 | SRR15026641 |
| MZ530993 | SRR15026639 |
| MZ530999 | SRR15026654 |
| MZ531000 | SRR15026643 |
| MZ531001 | SRR15026638 |
| MZ531003 | SRR15026153 |
| MZ531005 | SRR15026049 |
| MZ531006 | SRR15026044 |
| MZ531007 | SRR15026030 |
| MZ531017 | SRR15026042 |
| MZ531019 | SRR15026149 |
| MZ531026 | SRR15026034 |
| MZ531034 | SRR15026543 |
| MZ531037 | SRR15026546 |
| MZ531041 | SRR15026483 |
| MZ531044 | SRR15026542 |
| MZ531047 | SRR15027001 |
| MZ531048 | SRR15026469 |
| MZ531052 | SRR15026473 |
| MZ531053 | SRR15026471 |
| MZ531057 | SRR15026540 |
| MZ531064 | SRR15026480 |
| MZ531065 | SRR15026487 |
| MZ531066 | SRR15026532 |
| MZ531069 | SRR15026476 |
| MZ531072 | SRR15026495 |
| MZ531073 | SRR15026481 |
| MZ531074 | SRR15026535 |
| MZ531075 | SRR15026537 |
| MZ531078 | SRR15026539 |
| MZ531083 | SRR15026010 |
| MZ531092 | SRR15026987 |
| MZ531093 | SRR15026986 |
| MZ531095 | SRR15026985 |
| MZ531098 | SRR15026995 |
| MZ531101 | SRR15026117 |
| MZ531107 | SRR15026663 |
| MZ531111 | SRR15026122 |
| MZ531113 | SRR15026981 |
| MZ531114 | SRR15026119 |
| MZ531120 | SRR15026979 |
| MZ531127 | SRR15026992 |
| MZ531128 | SRR15026976 |
| MZ531129 | SRR15026123 |
| MZ531133 | SRR15026996 |
| MZ531135 | SRR15026561 |
| MZ531137 | SRR15026017 |
| MZ531138 | SRR15026016 |
| MZ531141 | SRR15026005 |
| MZ531146 | SRR15026660 |
| MZ531156 | SRR15026224 |
| MZ531157 | SRR15026212 |
| MZ531166 | SRR15026222 |
| MZ531187 | SRR15026233 |
| MZ531195 | SRR15026137 |
| MZ531204 | SRR15026700 |
| MZ531205 | SRR15026701 |
| MZ531208 | SRR15026691 |
| MZ531210 | SRR15026307 |
| MZ531211 | SRR15026702 |
| MZ531212 | SRR15026693 |
| MZ531215 | SRR15026686 |
| MZ531216 | SRR15026688 |
| MZ531223 | SRR15026276 |
| MZ531225 | SRR15026178 |
| MZ531234 | SRR15026171 |
| MZ531236 | SRR15026297 |
| MZ531238 | SRR15026305 |
| MZ531240 | SRR15026908 |
| MZ531241 | SRR15026692 |
| MZ531245 | SRR15026710 |
| MZ531249 | SRR15026288 |
| MZ531251 | SRR15026697 |
| MZ531252 | SRR15026696 |
| MZ531255 | SRR15026304 |
| MZ531256 | SRR15026287 |
| MZ531258 | SRR15026281 |
| MZ531260 | SRR15026296 |
| MZ531261 | SRR15026397 |
| MZ531270 | SRR15026373 |
| MZ531273 | SRR15026376 |
| MZ531280 | SRR15026374 |
| MZ531284 | SRR15026382 |
| MZ531285 | SRR15026920 |
| MZ531290 | SRR15026191 |
| MZ531291 | SRR15026192 |
| MZ531293 | SRR15026188 |
| MZ531294 | SRR15026197 |
| MZ531300 | SRR15026185 |
| MZ531301 | SRR15026914 |
| MZ531302 | SRR15026190 |
| MZ531303 | SRR15026919 |
| MZ531305 | SRR15026187 |
| MZ531306 | SRR15026184 |
| MZ531309 | SRR15026929 |
| MZ531311 | SRR15026077 |
| MZ531317 | SRR15026918 |
| MZ531319 | SRR15026198 |
| MZ531320 | SRR15026372 |
| MZ531328 | SRR15026613 |
| MZ531329 | SRR15026259 |
| MZ531330 | SRR15026258 |
| MZ531339 | SRR15026910 |
| MZ531341 | SRR15026921 |
| MZ531343 | SRR15026608 |
| MZ531346 | SRR15026078 |
| MZ531347 | SRR15026080 |
| MZ531350 | SRR15026162 |
| MZ531351 | SRR15026932 |
| MZ531352 | SRR15026933 |
| MZ531353 | SRR15026935 |
| MZ531355 | SRR15026082 |
| MZ531363 | SRR15026183 |
| MZ531365 | SRR15026180 |
| MZ531366 | SRR15026076 |
| MZ531368 | SRR15026915 |
| MZ531371 | SRR15026924 |
| MZ531372 | SRR15026917 |
| MZ531378 | SRR15026518 |
| MZ531380 | SRR15026516 |
| MZ531381 | SRR15026530 |
| MZ531384 | SRR15026249 |
| MZ531391 | SRR15026263 |
| MZ531394 | SRR15026520 |
| MZ531396 | SRR15026514 |
| MZ531397 | SRR15026525 |
| MZ531403 | SRR15026203 |
| MZ531405 | SRR15026316 |
| MZ531408 | SRR15026273 |
| MZ531412 | SRR15026602 |
| MZ531414 | SRR15026524 |
| MZ531419 | SRR15026508 |
| MZ531420 | SRR15026511 |
| MZ531426 | SRR15026503 |
| MZ531429 | SRR15026255 |
| MZ531436 | SRR15026510 |
| MZ531443 | SRR15026598 |
| MZ531449 | SRR15026319 |
| MZ531454 | SRR15026596 |
| MZ531456 | SRR15026321 |
| MZ531457 | SRR15026323 |
| MZ531464 | SRR15026512 |
| MZ531468 | SRR15026622 |
| MZ531469 | SRR15026624 |
| MZ531470 | SRR15026067 |
| MZ531471 | SRR15026066 |
| MZ531472 | SRR15026266 |
| MZ531474 | SRR15026623 |
| MZ531475 | SRR15026626 |
| MZ531476 | SRR15026617 |
| MZ531479 | SRR15026313 |
| MZ531483 | SRR15026625 |
| MZ531484 | SRR15026330 |
| MZ531485 | SRR15026068 |
| MZ531489 | SRR15026329 |
| MZ531493 | SRR15026058 |
| MZ531499 | SRR15026616 |
| MZ531510 | SRR15026092 |
| MZ531511 | SRR15026102 |
| MZ531512 | SRR15026088 |
| MZ531516 | SRR15026098 |
| MZ531517 | SRR15026094 |
| MZ531519 | SRR15026096 |
| MZ531521 | SRR15026089 |
| MZ531523 | SRR15026093 |
| MZ531531 | SRR15026361 |
| MZ531535 | SRR15026465 |
| MZ531538 | SRR15026464 |
| MZ531550 | SRR15026444 |
| MZ531552 | SRR15026441 |
| MZ531558 | SRR15026366 |
| MZ531561 | SRR15026868 |
| MZ531562 | SRR15026445 |
| MZ531563 | SRR15026458 |
| MZ531565 | SRR15026363 |
| MZ531566 | SRR15026367 |
| MZ531569 | SRR15026370 |
| MZ531570 | SRR15026436 |
| MZ531572 | SRR15026845 |
| MZ531574 | SRR15026839 |
| MZ531578 | SRR15026833 |
| MZ531581 | SRR15026835 |
| MZ531583 | SRR15026834 |
| MZ531588 | SRR15026836 |
| MZ531590 | SRR15026352 |
| MZ531591 | SRR15026940 |
| MZ531593 | SRR15026354 |
| MZ531594 | SRR15026355 |
| MZ531597 | SRR15026853 |
| MZ531603 | SRR15026864 |
| MZ531604 | SRR15026858 |
| MZ531605 | SRR15026857 |
| MZ531608 | SRR15026772 |
| MZ531615 | SRR15026770 |
| MZ531616 | SRR15026844 |
| MZ531618 | SRR15026778 |
| MZ531619 | SRR15026759 |
| MZ531621 | SRR15026755 |
| MZ531627 | SRR15026758 |
| MZ531629 | SRR15026111 |
| MZ531645 | SRR15026846 |
| MZ531647 | SRR15026969 |
| MZ531651 | SRR15026819 |
| MZ531652 | SRR15026832 |
| MZ531657 | SRR15026955 |
| MZ531667 | SRR15026964 |
| MZ531678 | SRR15026115 |
| MZ531700 | SRR15026740 |
| MZ531703 | SRR15026720 |
| MZ531705 | SRR15026729 |
| MZ531707 | SRR15026726 |
| MZ531716 | SRR15026716 |
| MZ531717 | SRR15026717 |
| MZ531723 | SRR15026948 |
| MZ531727 | SRR15026674 |
| MZ531732 | SRR15026677 |
| MZ531734 | SRR15026679 |

Table S.5 list of Alpha strains with SMRT data (third generation sequence data)

| Sequence Accession ID | SRA Accession ID |
| --- | --- |
| OM344484 | SRR20521394 |
| OM225458 | SRR21256058 |
| OM227298 | SRR21253440 |
| OM174384 | SRR21264618 |
| OM174633 | SRR21264430 |
| OM174823 | SRR21264528 |
| OM175640 | SRR21265426 |
| OM176028 | SRR21265046 |
| OM120692 | SRR21260208 |
| OM120946 | SRR21259957 |
| OM122135 | SRR21261676 |
| OM122524 | SRR21261165 |
| OM122604 | SRR21261881 |
| OL915294 | SRR20562139 |
| OL915434 | SRR20559553 |
| OL915779 | SRR20560484 |
| OL916290 | SRR20559645 |
| OL916457 | SRR20560828 |
| OL917051 | SRR20561229 |
| OL917159 | SRR20560208 |
| OL917545 | SRR20562626 |
| OL917639 | SRR20560920 |
| OL917703 | SRR20561774 |
| OL917818 | SRR20562120 |
| OL918594 | SRR20560517 |
| OL918595 | SRR20561026 |
| OL918814 | SRR20563200 |
| OL881682 | SRR20559042 |
| OL881807 | SRR20558989 |
| OL882218 | SRR20558577 |
| OL882385 | SRR20559183 |
| OL882743 | SRR20557998 |
| OL883180 | SRR20557765 |
| OL883993 | SRR20558760 |
| OL641731 | SRR20587864 |
| OL558398 | SRR20624410 |
| OL558410 | SRR20623523 |
| MZ848648 | SRR15233177 |
| MZ848657 | SRR15233103 |
| MZ848661 | SRR15231966 |
| MZ848670 | SRR15231973 |
| MZ848675 | SRR15233645 |
| MZ848681 | SRR15231222 |
| MZ848696 | SRR15232174 |
| MZ848697 | SRR15231020 |
| MZ848700 | SRR15231015 |
| MZ848701 | SRR15231211 |
| MZ848705 | SRR15231571 |
| MZ848710 | SRR15231140 |
| MZ848719 | SRR15231740 |
| MZ848729 | SRR15233805 |
| MZ848809 | SRR15231272 |
| MZ848837 | SRR15231268 |
| MZ848967 | SRR15233704 |
| MZ848991 | SRR15233182 |
| MZ848996 | SRR15233583 |
| MZ849031 | SRR15233221 |
| MZ849039 | SRR15233176 |
| MZ849046 | SRR15233566 |
| MZ849055 | SRR15233685 |
| MZ849056 | SRR15233590 |
| MZ849094 | SRR15231847 |
| MZ849140 | SRR15232563 |
| MZ849147 | SRR15232274 |
| MZ849153 | SRR15230870 |
| MZ849165 | SRR15233105 |
| MZ849172 | SRR15230905 |
| MZ849173 | SRR15233694 |
| MZ849178 | SRR15230902 |
| MZ849249 | SRR15231442 |
| MZ849254 | SRR15231468 |
| MZ849275 | SRR15233239 |
| MZ849295 | SRR15230915 |
| MZ849318 | SRR15231450 |
| MZ849325 | SRR15231955 |
| MZ849344 | SRR15231787 |
| MZ849370 | SRR15232905 |
| MZ849371 | SRR15232907 |
| MZ849393 | SRR15231240 |
| MZ849394 | SRR15231232 |
| MZ849424 | SRR15233641 |
| MZ849447 | SRR15232101 |
| MZ849459 | SRR15232076 |
| MZ849473 | SRR15233627 |
| MZ849491 | SRR15233497 |
| MZ849494 | SRR15231733 |
| MZ849517 | SRR15232926 |
| MZ849521 | SRR15231726 |
| MZ849528 | SRR15231721 |
| MZ849554 | SRR15231154 |
| MZ849750 | SRR15231713 |
| MZ849770 | SRR15232185 |
| MZ849943 | SRR15231212 |
| MZ849973 | SRR15231208 |
| MZ849990 | SRR15231871 |
| MZ849994 | SRR15231012 |
| MZ850084 | SRR15231927 |
| MZ850134 | SRR15231918 |
| MZ850163 | SRR15231367 |
| MZ850231 | SRR15233738 |
| MZ850255 | SRR15231628 |
| MZ850277 | SRR15233323 |
| MZ850315 | SRR15232202 |
| MZ850348 | SRR15232232 |
| MZ850350 | SRR15232218 |
| MZ850401 | SRR15231923 |
| MZ850414 | SRR15233082 |
| MZ850922 | SRR15233391 |
| MZ851057 | SRR15232629 |
| MZ783188 | SRR15378575 |
| MZ783206 | SRR15382187 |
| MZ783207 | SRR15380991 |
| MZ783214 | SRR15379736 |
| MZ783241 | SRR15378956 |
| MZ783242 | SRR15378739 |
| MZ783244 | SRR15379637 |
| MZ783254 | SRR15382656 |
| MZ783255 | SRR15382653 |
| MZ783260 | SRR15378094 |
| MZ783265 | SRR15381514 |
| MZ783270 | SRR15380952 |
| MZ783274 | SRR15381190 |
| MZ783279 | SRR15380787 |
| MZ783281 | SRR15382002 |
| MZ783286 | SRR15380534 |
| MZ783314 | SRR15379703 |
| MZ783786 | SRR15380428 |
| MZ783790 | SRR15378592 |
| MZ783877 | SRR15379228 |
| MZ783934 | SRR15381748 |
| MZ784335 | SRR15382431 |
| MZ784782 | SRR15380058 |
| MZ784783 | SRR15379437 |
| MZ784812 | SRR15379665 |
| MZ784835 | SRR15381264 |
| MZ784910 | SRR15379869 |
| MZ784963 | SRR15379861 |
| MZ784964 | SRR15379874 |
| MZ784988 | SRR15379678 |
| MZ784993 | SRR15378782 |
| MZ784994 | SRR15378762 |
| MZ785095 | SRR15379884 |
| MZ785156 | SRR15379886 |
| MZ785157 | SRR15379887 |
| MZ785351 | SRR15381376 |
| MZ785389 | SRR15381115 |
| MZ785411 | SRR15379035 |
| MZ785423 | SRR15378401 |
| MZ785474 | SRR15382556 |
| MZ785495 | SRR15379729 |
| MZ785510 | SRR15381107 |
| MZ785539 | SRR15379288 |
| MZ785706 | SRR15378274 |
| MZ785918 | SRR15379990 |
| MZ785919 | SRR15379777 |
| MZ785982 | SRR15378923 |
| MZ786026 | SRR15380265 |
| MZ786041 | SRR15379078 |
| MZ786069 | SRR15379596 |
| MZ786102 | SRR15378702 |
| MZ786134 | SRR15379370 |
| MZ786157 | SRR15379382 |
| MZ786230 | SRR15379087 |
| MZ786263 | SRR15381227 |
| MZ786281 | SRR15378944 |
| MZ786318 | SRR15381215 |
| MZ786326 | SRR15378473 |
| MZ786331 | SRR15382166 |
| MZ786334 | SRR15382150 |
| MZ786344 | SRR15378470 |
| MZ786362 | SRR15378461 |
| MZ786425 | SRR15378736 |
| MZ786443 | SRR15380616 |
| MZ786444 | SRR15380296 |
| MZ786445 | SRR15378955 |
| MZ786450 | SRR15381862 |
| MZ786461 | SRR15381863 |
| MZ786484 | SRR15379115 |
| MZ786522 | SRR15378973 |
| MZ786538 | SRR15379628 |
| MZ786545 | SRR15380308 |
| MZ786550 | SRR15379634 |
| MZ786551 | SRR15379626 |
| MZ786563 | SRR15380311 |
| MZ786564 | SRR15382039 |
| MZ786575 | SRR15380032 |
| MZ786584 | SRR15382665 |
| MZ786595 | SRR15382035 |
| MZ786622 | SRR15382670 |
| MZ786694 | SRR15378751 |
| MZ786700 | SRR15380019 |
| MZ786717 | SRR15381510 |
| MZ786739 | SRR15381690 |
| MZ786748 | SRR15381691 |
| MZ786759 | SRR15378752 |
| MZ786767 | SRR15381505 |
| MZ786777 | SRR15381872 |
| MZ786786 | SRR15379645 |
| MZ786825 | SRR15378641 |
| MZ786847 | SRR15382349 |
| MZ786864 | SRR15381421 |
| MZ786876 | SRR15378665 |
| MZ786879 | SRR15378438 |
| MZ786905 | SRR15381204 |
| MZ786948 | SRR15378661 |
| MZ786957 | SRR15378420 |
| MZ786987 | SRR15378653 |
| MZ787005 | SRR15381196 |
| MZ787021 | SRR15379357 |
| MZ787058 | SRR15378913 |
| MZ787065 | SRR15381828 |
| MZ787066 | SRR15382012 |
| MZ787069 | SRR15382791 |
| MZ787072 | SRR15381838 |
| MZ787096 | SRR15382799 |
| MZ787115 | SRR15380795 |
| MZ787121 | SRR15378052 |
| MZ787237 | SRR15379578 |
| MZ787257 | SRR15379359 |
| MZ787276 | SRR15379295 |
| MZ787296 | SRR15382007 |
| MZ787363 | SRR15379188 |
| MZ787378 | SRR15379197 |
| MZ787413 | SRR15380474 |
| MZ787425 | SRR15380491 |
| MZ787426 | SRR15380484 |
| MZ787427 | SRR15380481 |
| MZ787428 | SRR15380690 |
| MZ787481 | SRR15380713 |
| MZ591356 | SRR15156709 |
| MZ591362 | SRR15156673 |
| MZ591397 | SRR15157454 |
| MZ591409 | SRR15155931 |
| MZ591415 | SRR15156390 |
| MZ591489 | SRR15156676 |
| MZ591498 | SRR15156682 |
| MZ591525 | SRR15156726 |
| MZ591535 | SRR15156731 |
| MZ591545 | SRR15156656 |
| MZ591580 | SRR15156642 |
| MZ591583 | SRR15156572 |
| MZ591586 | SRR15156569 |
| MZ591587 | SRR15156552 |
| MZ591614 | SRR15157144 |
| MZ591616 | SRR15156626 |
| MZ591635 | SRR15156646 |
| MZ591732 | SRR15157083 |
| MZ591830 | SRR15156948 |
| MZ591835 | SRR15156951 |
| MZ592144 | SRR15156866 |
| MZ592194 | SRR15156916 |
| MZ592197 | SRR15156209 |
| MZ592234 | SRR15157247 |
| MZ592382 | SRR15156082 |
| MZ592656 | SRR15156278 |
| MZ592762 | SRR15156371 |
| MZ553974 | SRR15088003 |
| MZ553993 | SRR15087753 |
| MZ554028 | SRR15087938 |
| MZ554029 | SRR15087958 |
| MZ554061 | SRR15087874 |
| MZ554067 | SRR15087822 |
| MZ554078 | SRR15087795 |
| MZ554100 | SRR15087846 |
| MZ554131 | SRR15087885 |
| MZ554133 | SRR15087995 |
| MZ554146 | SRR15087818 |
| MZ554228 | SRR15088233 |
| MZ554233 | SRR15088232 |
| MZ554275 | SRR15088291 |
| MZ554294 | SRR15088259 |
| MZ554310 | SRR15088274 |
| MZ554325 | SRR15088355 |
| MZ554369 | SRR15088360 |
| MZ555277 | SRR15088508 |
| MZ555422 | SRR15088995 |
| MZ555456 | SRR15088990 |
| MZ555458 | SRR15088955 |
| MZ530868 | SRR15026878 |
| MZ530872 | SRR15026795 |
| MZ530922 | SRR15026564 |
| MZ530932 | SRR15026572 |
| MZ531068 | SRR15026479 |
| MZ531227 | SRR15026179 |
| MZ531248 | SRR15026285 |
| MZ531335 | SRR15026934 |
| MZ531340 | SRR15026922 |
| MZ531349 | SRR15026253 |
| MZ531389 | SRR15026244 |
| MZ531451 | SRR15026204 |

Table S.6 list of Delta strains with SMRT data (third generation sequence data)

| Sequence Accession ID | SRA Accession ID |
| --- | --- |
| OQ432276 | SRR23452416 |
| OQ421293 | SRR23404123 |
| OQ421298 | SRR23404161 |
| OQ421355 | SRR23404149 |
| OQ352311 | SRR23253710 |
| OQ352319 | SRR23253725 |
| OQ352326 | SRR23253697 |
| OQ352356 | SRR23253670 |
| OQ352404 | SRR23253609 |
| OQ345091 | SRR23258590 |
| OQ345229 | SRR23258529 |
| OQ294208 | SRR23144371 |
| OQ294411 | SRR23145354 |
| OQ187097 | SRR23002528 |
| OQ187260 | SRR23002347 |
| OQ187438 | SRR23002614 |
| OQ187619 | SRR23002505 |
| OQ165346 | SRR22976449 |
| OQ165577 | SRR22975995 |
| OQ165748 | SRR22978039 |
| OQ165872 | SRR22975977 |
| OQ166000 | SRR22977072 |
| OQ166099 | SRR22977769 |
| OQ166101 | SRR22977464 |
| OQ166516 | SRR22978234 |
| OQ167360 | SRR22976273 |
| OQ122220 | SRR22902706 |
| OQ122510 | SRR22903563 |
| OQ122904 | SRR22902537 |
| OQ122952 | SRR22903258 |
| OQ122958 | SRR22902501 |
| OQ123013 | SRR22903199 |
| OP968283 | SRR22583578 |
| OP968291 | SRR22583842 |
| OP968331 | SRR22584099 |
| OP968342 | SRR22583513 |
| OP968365 | SRR22584183 |
| OP968395 | SRR22584263 |
| OP968480 | SRR22584179 |
| OP968580 | SRR22584334 |
| OP968625 | SRR22584317 |
| OP968628 | SRR22584072 |
| OP968688 | SRR22584399 |
| OP968693 | SRR22584333 |
| OP968817 | SRR22584124 |
| OP968901 | SRR22584229 |
| OP968917 | SRR22583875 |
| OP954944 | SRR22555360 |
| OP954977 | SRR22555684 |
| OP955134 | SRR22555938 |
| OP955174 | SRR22555515 |
| OP955215 | SRR22555548 |
| OP955312 | SRR22555740 |
| OP955315 | SRR22555718 |
| OP955476 | SRR22555617 |
| OP955517 | SRR22556006 |
| OP926057 | SRR22493993 |
| OP926059 | SRR22494169 |
| OP926063 | SRR22493992 |
| OP926107 | SRR22493823 |
| OP926133 | SRR22493880 |
| OP926152 | SRR22493513 |
| OP926206 | SRR22494055 |
| OP926245 | SRR22493516 |
| OP926286 | SRR22494009 |
| OP926288 | SRR22494010 |
| OP926289 | SRR22494008 |
| OP926299 | SRR22493902 |
| OP926329 | SRR22494182 |
| OP926348 | SRR22494229 |
| OP926349 | SRR22493577 |
| OP926350 | SRR22493578 |
| OP926367 | SRR22493636 |
| OP926371 | SRR22493580 |
| OP926407 | SRR22493506 |
| OP926410 | SRR22494247 |
| OP926428 | SRR22493801 |
| OP926475 | SRR22493934 |
| OP926531 | SRR22493811 |
| OP926557 | SRR22494274 |
| OP926570 | SRR22494271 |
| OP926594 | SRR22494092 |
| OP926595 | SRR22493884 |
| OP926660 | SRR22493588 |
| OP926716 | SRR22493688 |
| OP926751 | SRR22494098 |
| OP926769 | SRR22493668 |
| OP926807 | SRR22493962 |
| OP926811 | SRR22493946 |
| OP923721 | SRR22475871 |
| OP923736 | SRR22475817 |
| OP923737 | SRR22475820 |
| OP923778 | SRR22475870 |
| OP923780 | SRR22475823 |
| OP923787 | SRR22475810 |
| OP923792 | SRR22475866 |
| OP923803 | SRR22475777 |
| OP923807 | SRR22475784 |
| OP923815 | SRR22475845 |
| OP923816 | SRR22475789 |
| OP923829 | SRR22475778 |
| OP906467 | SRR22451864 |
| OP906512 | SRR22452183 |
| OP906517 | SRR22451674 |
| OP906527 | SRR22451721 |
| OP906531 | SRR22452182 |
| OP906534 | SRR22451848 |
| OP906569 | SRR22452189 |
| OP906574 | SRR22452187 |
| OP906575 | SRR22450931 |
| OP906615 | SRR22452160 |
| OP906625 | SRR22451657 |
| OP906636 | SRR22450956 |
| OP906638 | SRR22451825 |
| OP906697 | SRR22451461 |
| OP906724 | SRR22450869 |
| OP906781 | SRR22452129 |
| OP906828 | SRR22451068 |
| OP906899 | SRR22450926 |
| OP906908 | SRR22451820 |
| OP907093 | SRR22452022 |
| OP907185 | SRR22452042 |
| OP907216 | SRR22452053 |
| OP907230 | SRR22452048 |
| OP907245 | SRR22451051 |
| OP907269 | SRR22451488 |
| OP907289 | SRR22451790 |
| OP907290 | SRR22451789 |
| OP907299 | SRR22451804 |
| OP907324 | SRR22451537 |
| OP907348 | SRR22451514 |
| OP907390 | SRR22450834 |
| OP907411 | SRR22451676 |
| OP907517 | SRR22450645 |
| OP907528 | SRR22451759 |
| OP907561 | SRR22451359 |
| OP907577 | SRR22451928 |
| OP907590 | SRR22451212 |
| OP907791 | SRR22451192 |
| OP907806 | SRR22450680 |
| OP907878 | SRR22451887 |
| OP907912 | SRR22451748 |
| OP907913 | SRR22451025 |
| OP907991 | SRR22452204 |
| OP882772 | SRR22399821 |
| OP882782 | SRR22400044 |
| OP882791 | SRR22400092 |
| OP882808 | SRR22400038 |
| OP882825 | SRR22399985 |
| OP882862 | SRR22400387 |
| OP882887 | SRR22400028 |
| OP882929 | SRR22400105 |
| OP882943 | SRR22399911 |
| OP883011 | SRR22399880 |
| OP883029 | SRR22399820 |
| OP883046 | SRR22400345 |
| OP883109 | SRR22399887 |
| OP883143 | SRR22399848 |
| OP883149 | SRR22399608 |
| OP883155 | SRR22399681 |
| OP883167 | SRR22399697 |
| OP883169 | SRR22400329 |
| OP883170 | SRR22399635 |
| OP883230 | SRR22399668 |
| OP883243 | SRR22400187 |
| OP883244 | SRR22400244 |
| OP883259 | SRR22400325 |
| OP883336 | SRR22400261 |
| OP883373 | SRR22399854 |
| OP883413 | SRR22399846 |
| OP883417 | SRR22400191 |
| OP883447 | SRR22400379 |
| OP883460 | SRR22400332 |
| OP883475 | SRR22400115 |
| OP883478 | SRR22400120 |
| OP875117 | SRR22383686 |
| OP875164 | SRR22385017 |
| OP875172 | SRR22385056 |
| OP875176 | SRR22383773 |
| OP875179 | SRR22384673 |
| OP875191 | SRR22383707 |
| OP875195 | SRR22383800 |
| OP875275 | SRR22383708 |
| OP875283 | SRR22383792 |
| OP875313 | SRR22384776 |
| OP875328 | SRR22383723 |
| OP875336 | SRR22383945 |
| OP875363 | SRR22384878 |
| OP875392 | SRR22383692 |
| OP875393 | SRR22383690 |
| OP875415 | SRR22384947 |
| OP875439 | SRR22383994 |
| OP875484 | SRR22384978 |
| OP875507 | SRR22384922 |
| OP875607 | SRR22384232 |
| OP875608 | SRR22384244 |
| OP875621 | SRR22383896 |
| OP875703 | SRR22384227 |
| OP875726 | SRR22384225 |
| OP875731 | SRR22384706 |
| OP875754 | SRR22384866 |
| OP875772 | SRR22384185 |
| OP875781 | SRR22384863 |
| OP875787 | SRR22384641 |
| OP875853 | SRR22384608 |
| OP875915 | SRR22384603 |
| OP875918 | SRR22384989 |
| OP875945 | SRR22384686 |
| OP875979 | SRR22383549 |
| OP876017 | SRR22384913 |
| OP876034 | SRR22383548 |
| OP876154 | SRR22384820 |
| OP876167 | SRR22384891 |
| OP876201 | SRR22384462 |
| OP876205 | SRR22384484 |
| OP876232 | SRR22383658 |
| OP876235 | SRR22384549 |
| OP876280 | SRR22383643 |
| OP876288 | SRR22384374 |
| OP876454 | SRR22384811 |
| OP876462 | SRR22384272 |
| OP876496 | SRR22384316 |
| OP876545 | SRR22384575 |
| OP876555 | SRR22384415 |
| OP876563 | SRR22384564 |
| OP876566 | SRR22384897 |
| OP876586 | SRR22384332 |
| OP876631 | SRR22384144 |
| OP876692 | SRR22385082 |
| OP876693 | SRR22385120 |
| OP861135 | SRR22349552 |
| OP861144 | SRR22349482 |
| OP861156 | SRR22349592 |
| OP861220 | SRR22349323 |
| OP861239 | SRR22349556 |
| OP861274 | SRR22349519 |
| OP861289 | SRR22349460 |
| OP861358 | SRR22349528 |
| OP854946 | SRR22342407 |
| OP854964 | SRR22342216 |
| OP854972 | SRR22342115 |
| OP855023 | SRR22342089 |
| OP855041 | SRR22342094 |
| OP855066 | SRR22342061 |
| OP855079 | SRR22342202 |
| OP855088 | SRR22342101 |
| OP855094 | SRR22342123 |
| OP855134 | SRR22342098 |
| OP855252 | SRR22342332 |
| OP827059 | SRR22305738 |
| OP827070 | SRR22305949 |
| OP827072 | SRR22305053 |
| OP827107 | SRR22305974 |
| OP827112 | SRR22305745 |
| OP827147 | SRR22305409 |
| OP827159 | SRR22305050 |
| OP827163 | SRR22305406 |
| OP827189 | SRR22305421 |
| OP827197 | SRR22305419 |
| OP827231 | SRR22305942 |
| OP827233 | SRR22305059 |
| OP827251 | SRR22305330 |
| OP827252 | SRR22305009 |
| OP827362 | SRR22305889 |
| OP827364 | SRR22305314 |
| OP827411 | SRR22305107 |
| OP827437 | SRR22304829 |
| OP827441 | SRR22305263 |
| OP827466 | SRR22305184 |
| OP827473 | SRR22305175 |
| OP827482 | SRR22305151 |
| OP827507 | SRR22305141 |
| OP827524 | SRR22305545 |
| OP827531 | SRR22305027 |
| OP827533 | SRR22305546 |
| OP827582 | SRR22305908 |
| OP827635 | SRR22305597 |
| OP827637 | SRR22305915 |
| OP827644 | SRR22305219 |
| OP827698 | SRR22305869 |
| OP827749 | SRR22305241 |
| OP827789 | SRR22305236 |
| OP827814 | SRR22305736 |
| OP827831 | SRR22305228 |
| OP827835 | SRR22305851 |
| OP827847 | SRR22305077 |
| OP827867 | SRR22305003 |
| OP827947 | SRR22304826 |
| OP827955 | SRR22304895 |
| OP827975 | SRR22306097 |
| OP827976 | SRR22305482 |
| OP827981 | SRR22304770 |
| OP827986 | SRR22306154 |
| OP828004 | SRR22306117 |
| OP828030 | SRR22305509 |
| OP828032 | SRR22305534 |
| OP828039 | SRR22305442 |
| OP828063 | SRR22305522 |
| OP828072 | SRR22305313 |
| OP828115 | SRR22304966 |
| OP828163 | SRR22305225 |
| OP828166 | SRR22305525 |
| OP828195 | SRR22305334 |
| OP828214 | SRR22306136 |
| OP828258 | SRR22304820 |
| OP828265 | SRR22306043 |
| OP828286 | SRR22305636 |
| OP828293 | SRR22305278 |
| OP828313 | SRR22305625 |
| OP828386 | SRR22304806 |
| OP828387 | SRR22304803 |
| OP828421 | SRR22306159 |
| OP828424 | SRR22306035 |
| OP828429 | SRR22306164 |
| OP828431 | SRR22304808 |
| OP823568 | SRR22297595 |
| OP823573 | SRR22297613 |
| OP823609 | SRR22297516 |
| OP823644 | SRR22297519 |
| OP823658 | SRR22297569 |
| OP824429 | SRR22291988 |
| OP824430 | SRR22291977 |
| OP824445 | SRR22291990 |
| OP824457 | SRR22291976 |
| OP824459 | SRR22291974 |
| OP824461 | SRR22291972 |
| OP824465 | SRR22291968 |
| OP824472 | SRR22291960 |
| OP808478 | SRR22264435 |
| OP808479 | SRR22264440 |
| OP808507 | SRR22264967 |
| OP808556 | SRR22265174 |
| OP808558 | SRR22265154 |
| OP808562 | SRR22264944 |
| OP808566 | SRR22265142 |
| OP808570 | SRR22265160 |
| OP808604 | SRR22265181 |
| OP808606 | SRR22264954 |
| OP808611 | SRR22264899 |
| OP808616 | SRR22264896 |
| OP808687 | SRR22265264 |
| OP808734 | SRR22265088 |
| OP808735 | SRR22265089 |
| OP808736 | SRR22264852 |
| OP808740 | SRR22265062 |
| OP808744 | SRR22265066 |
| OP808745 | SRR22264870 |
| OP808747 | SRR22264538 |
| OP808749 | SRR22265079 |
| OP808810 | SRR22264909 |
| OP808825 | SRR22265085 |
| OP808829 | SRR22264450 |
| OP808854 | SRR22264725 |
| OP808875 | SRR22265388 |
| OP808898 | SRR22265383 |
| OP808903 | SRR22265145 |
| OP808914 | SRR22264342 |
| OP808929 | SRR22264573 |
| OP808951 | SRR22264856 |
| OP808971 | SRR22265077 |
| OP808973 | SRR22264583 |
| OP808991 | SRR22264532 |
| OP809001 | SRR22264845 |
| OP809022 | SRR22265191 |
| OP809041 | SRR22264750 |
| OP809095 | SRR22264755 |
| OP809178 | SRR22264835 |
| OP809184 | SRR22265009 |
| OP809228 | SRR22264842 |
| OP809249 | SRR22264772 |
| OP809251 | SRR22264790 |
| OP809256 | SRR22264791 |
| OP809291 | SRR22264457 |
| OP809330 | SRR22264994 |
| OP809369 | SRR22264730 |
| OP809395 | SRR22264392 |
| OP809401 | SRR22265318 |
| OP809410 | SRR22264737 |
| OP809436 | SRR22264924 |
| OP809463 | SRR22265321 |
| OP809480 | SRR22264680 |
| OP809481 | SRR22264681 |
| OP809523 | SRR22264509 |
| OP809540 | SRR22264468 |

Table S.7 list of Omicron strains with SMRT data (third generation sequence data)
